# Well-posedness and stability analysis of an epidemic model with infection age and spatial diffusion

**DOI:** 10.1007/s00285-023-01980-y

**Published:** 2023-08-31

**Authors:** Christoph Walker

**Affiliations:** Institut für Angewandte Mathematik, Leibniz Universität Hannover, Welfengarten 1, 30167 Hannover, Germany

**Keywords:** Age-strucutre, Spatial diffusion, Stability of steady states, 35M10, 47D06, 92D30, 47A10

## Abstract

A compartment epidemic model for infectious disease spreading is investigated, where movement of individuals is governed by spatial diffusion. The model includes infection age of the infected individuals and assumes a logistic growth of the susceptibles. Global well-posedness of the equations within the class of nonnegative smooth solutions is shown. Moreover, spectral properties of the linearization around a steady state are derived. This yields the notion of linear stability which is used to determine stability properties of the disease-free and the endemic steady state.

## Introduction

We consider a compartment epidemic model for infectious disease spreading. The total population is divided into susceptible and infected individuals which move in space by diffusion. Infectious individuals are structured by infective age keeping track of the time elapsed since an individual first acquires the disease. A logistic growth is assumed for the susceptibles.

Let *S*(*t*, *x*) and *I*(*t*, *a*, *x*) be the densities of susceptible and infected individuals, respectively, at time $t\ge 0$, position $x\in \Omega$, and infection age $a\in (0,a_m)$, where $\Omega \subset \mathbb {R}^n$ with $n\le 3$ is a bounded, smooth domain, and $a_m\in [0,\infty )$ is the maximal invective age. The population of susceptible individuals is assumed to obey a logistic growth with intrinsic growth rate $\kappa _1>0$ and carrying capacity $\kappa _2>0$. Susceptible individuals are infected at a rate *b*(*a*, *x*) by infected individuals of invective age *a* and position *x*. Infectious individuals die naturally and disease-induced at a combined rate $\mu (a,x)\ge 0$. They may recover and enter directly the class of susceptibles at a rate $r(a,x)\ge 0$. We shall thus focus on the equations

1.1a $$\begin{aligned} \partial _t S(t,x)&=d_1\Delta S(t,x)+\kappa _1\left( 1-\dfrac{1}{\kappa _2}S(t,x)\right) S(t,x) \nonumber \\&\qquad -S(t,x)\int _0^{a_m} b(a,x)\,I(t,a,x)\,\textrm{d}a +\int _0^{a_m} r(a,x)\,I(t,a,x)\,\textrm{d}a\,, \end{aligned}$$

1.1b $$\begin{aligned} D I(t,a,x)&=d(a)\Delta I(t,a,x)-m(a,x)I(t,a,x)-r(a,x)I(t,a,x)\,, \end{aligned}$$

1.1c $$\begin{aligned} I(t,0,x)&=S(t,x)\int _0^{a_m} b(a,x)\,I(t,a,x)\,\textrm{d}a\,, \end{aligned}$$

for $t>0$, $a\in (0,a_m)$, and $x\in \Omega$. The differentiation operator *D* in (1.1b) is defined as

$$\begin{aligned} D I(t,a,\cdot ):=\lim _{h\rightarrow 0^+} \frac{1}{h}\big (I(t+h,a+h,\cdot )-I(t,a,\cdot )\big ) \end{aligned}$$

and is thus, if *I* is continuously differentiable with respect to *t* and *a*, given by

$$\begin{aligned} DI(t,a,\cdot )=\partial _t I(t,a,\cdot )+\partial _a I(t,a,\cdot ). \end{aligned}$$

For notational simplicity we will take a susceptible diffusion rate $d_1=1$ and consider a diffusion coefficient $d=d(a)>0$ for infected individuals dependent only upon infection age (though a space dependence does not alter the subsequent results). The equations are supplemented by the initial conditions

1.1d $$\begin{aligned} S(0,x)=S_0(x)\,,\quad I(0,a,x)&= I_0(a,x)\,,\qquad (a,x)\in (0,a_m)\times \Omega \,, \end{aligned}$$

and the boundary conditions

1.1e $$\begin{aligned} (1-\delta )S(t,x)+\delta \partial _\nu S(t,x)= 0\,,\qquad (1-\delta )I(t,a,x)+\delta \partial _\nu I(t,a,x)&= 0 \end{aligned}$$

for $(t,a,x)\in (0,\infty )\times (0,a_m)\times \partial \Omega$. Here, $\delta \in \{0,1\}$ is fixed so that $\delta =0$ corresponds to Dirichlet boundary conditions, while $\delta =1$ yields Neumann boundary conditions with $\partial _\nu I=\nabla I\cdot \nu$ denoting the derivative in normal direction $\nu$ on the boundary $\partial \Omega$ (we treat the two cases simultaneously).

Age-structured compartment epidemic models and age-structured population models in general have been investigated since many years (Thieme (2003); Webb (1985, 2008)). The particular case of equations (1.1) without spatial diffusion (and $r=0$) was studied in Cao et al. (2021). Therein, criteria for stability and instability of the disease-free and the endemic steady states were obtained in dependence on the corresponding basic reproduction number. Moreover, conditions for the occurrence of Hopf bifurcation were presented.

The inclusion of spatial heterogeneity in age-structured populations leads to additional technical difficulties in the analysis. We refer to the monograph of Webb (2008) for a general treatment of and a comprehensive overview on such problems. Regarding SIS- and SIR-models there are various linear and nonlinear variants involving age and spatial structure with Laplace diffusion. The list includes the pioneering works of Webb (1980, 1981, 1982) on epidemic models including incubation periods, followed by Fitzgibbon et al. (1995, 1996), Kubo and Langlais (1994), Langlais and Busenberg (1997) and, more recently, Chekroun and Kuniya (2019, 2020a, 2020b); Di Blasio (2010); Ducrot and Magal (2009); Ducrot et al. (2010); Ducrot and Magal (2011); Kim (2006); Kuniya and Oizumi (2015) though these references are non-exhaustive. The cited papers address various questions under different modeling hypotheses, for instance related to well-posedness of the equations, existence and stability properties of disease-free and endemic steady states, disease persistence, traveling wave solutions, or numerical simulations. We also refer to Kang and Ruan (2021) and the references therein for age-structured epidemic models describing long-distance spreading of diseases by nonlocal diffusion.

In this research we shall focus on the particular model (1.1). We first prove the existence and uniqueness of positive, global, smooth solutions by using a semigroup approach relying on results outlined in Webb (2008). We then show that the linearization of these equations around a steady state yields a strongly continuous semigroup, and we use the spectrum of its generator for determining linear stability properties of steady states. Without further assumptions we prove stability or instability of the disease-free steady state in dependence on the reproduction number. In a particular case of (1.1) assuming spatially homogeneous rates and Neumann boundary conditions we improve the local stability of this steady state to global stability. Moreover, we investigate the stability of the endemic steady state.

In the following Sect. 2 we present our main results. Section 3 is dedicated to the proof of the well-posedness of (1.1). The details on the linearized problem in the general case are given in Sect. 4 and then applied in Sect. 5. The application to a simplified version of equations (1.1) with Neumann boundary conditions and spatially homogeneous rates are presented in Sect. 6. Some technical results are postponed to the Appendix 1.

## Main results

We first state the result on the well-posedness of (1.1) and then investigate linearized stability of steady states.

### Well-posedness

In order to present our existence result, we set $J:=[0,a_m]$ and take without loss of generality $d_1=1$. We assume that

2.1a $$\begin{aligned} \kappa _1\,,\kappa _2>0 \end{aligned}$$

and

2.1b $$\begin{aligned} d\in C^\rho (J)\,,\qquad d(a)\ge {\underline{d}}>0\,,\quad a\in J\,, \end{aligned}$$

for some $\rho >0$. Moreover,

2.1c $$\begin{aligned} m\in C^\rho \big (J,L_\infty (\Omega )\big )\,,\quad r\in C^\rho \big (J,L_\infty (\Omega )\big )\cap L_\infty \big (J,C^1({{\bar{\Omega }}})\big )\,,\qquad m,r\ge 0\,, \end{aligned}$$

and

2.1d $$\begin{aligned} b\in L_\infty \big (J,C^1({{\bar{\Omega }}})\big )\,, \quad b\ge 0\,,\quad b\not \equiv 0\,. \end{aligned}$$

Here, $C^\rho$ stands for $\rho$-Hölder continuous functions. The regularity assumptions on the data are mainly imposed in order to derive smooth solutions. Denote $\mathbb {R}^+:=[0,\infty )$ and ${\dot{\mathbb {R}}}^+:=(0,\infty )$.

We shall prove the following result on the existence, uniqueness, and regularity of global, positive solutions to (1.1):

#### Theorem 2.1

Assume (2.1) and $p\in \big (\max \{3n/4,2\},\infty \big )$. Then, given initial values $S_0\in L_p^+(\Omega )$ and $I_0\in L_1\big (J,L_p^+(\Omega )\big )$, there is a unique positive global solution (*S*, *I*) to (1.1) such that

$$\begin{aligned} S\in C\big (\mathbb {R}^+,L_p^+(\Omega )\big )\cap C^1\big ({\dot{\mathbb {R}}}^+,L_p(\Omega )\big )\cap C\big ({\dot{\mathbb {R}}}^+,W_p^2(\Omega )\big ) \end{aligned}$$

is a strong solution to (1.1a), while

$$\begin{aligned} I\in C\big (\mathbb {R}^+,L_1(J,L_p^+(\Omega ))\big ) \cap C\big ({\dot{\mathbb {R}}}^+,L_1(J,W_{p}^{2}(\Omega ))\big ) \end{aligned}$$

satisfies (1.1b) in the sense that

$$\begin{aligned} D I(t,a)=\big (d(a)\Delta -m(a,\cdot ) -r(a,\cdot )\big )I(t,a,\cdot )\ \text { in }\ L_p(\Omega ) \end{aligned}$$

for $t>0$ and a.e. $a\in (0,a_m)$. In fact, the solution map $(t,(S_0,I_0))\mapsto (S,I)(t)$ defines a global semiflow on $L_p^+(\Omega )\times L_1(J,L_p^+(\Omega ))$.

The proof of Theorem 2.1 is based on a semigroup representation of solutions in the spirit of Webb (2008) and on Banach’s fixed point theorem. It is performed in several steps in Sect. 3. In fact, it is not restricted to the particular nonlinearities in (1.1) and may rather be a template for similar problems. That the solution map defines a global semiflow paves the way to consider qualitative aspects of the model.

### Linearization around steady states

Assume (2.1) and fix an arbitrary steady state $(S_*,I_*)$ to (1.1), i.e. a time-independent solution. The regularizing effects of the Laplacian implies that we may assume without loss of generality the regularity

2.2 $$\begin{aligned} \begin{aligned}&S_*\in W_{p}^{2}(\Omega ), \quad S_*>0 \ \text { in }\ \Omega ,\\&I_*\in L_1\big (J,W_{p}^{2}(\Omega )\big )\cap W_1^1\big (J,L_p(\Omega )\big ),\quad I_*\ge 0 \ \text { in }\ J\times \Omega , \end{aligned} \end{aligned}$$

for some $p>n$. The linearization of (1.1) around the steady state $(S_*,I_*)$ is

2.3a $$\begin{aligned} \partial _t S(t,x)&=\Delta S(t,x)\!+\!\kappa _1S(t,x) - \dfrac{2\kappa _1 S_*(x)}{\kappa _2} S(t,x)\!-\!S(t,x)\int _0^{a_m} b(a,x)\,I_*(a,x)\,\textrm{d}a \nonumber \\&\quad -S_*(x)\int _0^{a_m} b(a,x)\,I(t,a,x)\,\textrm{d}a +\int _0^{a_m} r(a,x)\,I(t,a,x)\,\textrm{d}a \,, \end{aligned}$$

2.3b $$\begin{aligned} D I(t,a,x)&=d(a)\Delta I(t,a,x)-\big (m(a,x)+r(a,x)\big ) I(t,a,x)\,, \end{aligned}$$

2.3c $$\begin{aligned} I(t,0,x)&=S_*(x)\int _0^{a_m} b(a,x)\,I(t,a,x)\,\textrm{d}a +S(t,x)\int _0^{a_m} b(a,x)\,I_*(a,x)\,\textrm{d}a\,, \end{aligned}$$

for $(t,a, x)\in \mathbb {R}^+\times (0,a_m)\times \Omega$, and subject to the initial conditions

2.3d $$\begin{aligned} S(0,x)=S_0(x)\,,\quad I(0,a,x)&= I_0(a,x)\,,\qquad (a,x)\in (0,a_m)\times \Omega \,, \end{aligned}$$

and boundary conditions

2.3e $$\begin{aligned} (1-\delta )S(t,x)+\delta \partial _\nu S(t,x)= 0\,,\qquad (1-\delta )I(t,a,x)+\delta \partial _\nu I(t,a,x)&= 0 \end{aligned}$$

for $(t,a,x)\in (0,\infty )\times (0,a_m)\times \partial \Omega$. We shall show that the solutions (*S*, *I*) to the linearized problem (2.3) are given by a strongly continuous semigroup on $L_p(\Omega )\times L_1(J,L_p(\Omega ))$ with compact resolvent:

#### Theorem 2.2

Suppose (2.1) and $p>(2\vee n)$. Let $(S_*,I_*)$ be a steady state to (1.1) satisfying (2.2). Then, the solution (*S*, *I*) to the linearized equation (2.3) is given as

$$\begin{aligned} (S,I)(t)=\mathbb {S}_*(t)(S_0,I_0),\quad t\ge 0, \end{aligned}$$

where $(\mathbb {S}_*(t))_{t\ge 0}$ is a strongly continuous semigroup on $L_p(\Omega )\times L_1(J,L_p(\Omega ))$. Its generator has a compact resolvent and thus a pure point spectrum without finite accumulation point.

Theorem 2.2 is a consequence of Theorem 4.2 and Corollary 4.3 from Sect. 4. There, we also present more precise information on the semigroup and its generator. Note that the semigroup $(\mathbb {S}_*(t))_{t\ge 0}$ lacks positivity and thus less information on the spectrum of its generator is available in general. We refer to Remark 4.6 for further details.

### Linear stability

Due to Theorem 2.2, one may characterize stability properties of steady states based on the linearization of (1.1) around these steady states. That linearized stability indeed determines the (asymptotic) stability of steady states in certain nonlinear population models including age- and spatial structure has recently been shown in Walker (2023) and Walker and Zehetbauer (2022). We refrain, however, to prove this for problem (1.1).

Herein, we shall just call a steady state $(S_*,I_*)$ *linearly stable* if the generator of the semigroup associated with the linearization (2.3) of (1.1) (given in Theorem 2.2) has a spectrum lying entirely in the half plane $\textrm{Re}\, \lambda <0$ while we call the steady state *linearly unstable* if there is a spectral point in the half plane $\textrm{Re}\, \lambda >0$ (see Definition 4.4 and Remark 4.6 below for more details in this regard).

We assume for simplicity that

2.4 $$\begin{aligned} r\equiv 0 . \end{aligned}$$

We provide a stability analysis in $L_p(\Omega )\times L_1(J,L_p(\Omega ))$ of the trivial and the disease-free steady states. To state precise results we introduce the principal eigenvalue $\mu _0$ of the Laplacian $-\Delta$ on $\Omega$ subject to either Dirichlet boundary conditions (hence $\mu _0>0$) or Neumann boundary conditions (hence $\mu _0=0$).

#### Theorem 2.3

Suppose (2.1), (2.4), and let $p>(2\vee n)$.

$$\mathbf{(a)}$$
The trivial steady state $(S_*,I_*)=(0,0)$ to (1.1) is linearly unstable in the space $L_p(\Omega )\times L_1(J,L_p(\Omega ))$ if $\kappa _1>\mu _0$ and linearly stable if $\kappa _1<\mu _0$.
$$\mathbf{(b)}$$
There is a disease-free steady state $(S_*,I_*)=({\tilde{S}}_*,0)$ to (1.1) with a smooth function ${\tilde{S}}_*>0$ if and only if $\kappa _1>\mu _0$. In this case, the disease-free steady state $(S_*,I_*)=({\tilde{S}}_*,0)$ is unique (and given by ${\tilde{S}}_*=\kappa _2$ for Neumann boundary conditions), and there is a number ${\textsf{R}}_0>0$ such that it is linearly stable in the space $\hbox {L}{_{p}}(\Omega )\times {{ L}}_1({{ J,L}}_{{ p}}(\Omega ))$ if ${\textsf{R}}_0<1$ and linearly unstable if ${\textsf{R}}_0>1$.
$$\mathbf{(c)}$$
Let $\kappa _1>\mu _0$. There is no endemic state $(S_*,I_*)$ to (1.1) with $S_*, I_*\ge 0$ and $I_*\not \equiv 0$ if ${\textsf{R}}_0\le 1$.

The proof of Theorem 2.3 is presented in Sect. 5. The reproduction number ${\textsf{R}}_0>0$ is defined in (5.3a) and corresponds to the spectral radius of a compact irreducible operator (defined in (5.3b)) depending on ${\tilde{S}}_*$. It is open whether there is an endemic state $(S_*,I_*)$ with $S_*, I_*\ge 0$ and $I_*\not \equiv 0$ if ${\textsf{R}}_0>1$ (see Remark 5.5 in this regard). However, the existence of an endemic steady state in case that ${\textsf{R}}_0>1$ is easily obtained when assuming Neumann boundary conditions and spatially homogeneous rates *m* and *b* as shown in the next subsection.

### Linear stability in a particular model with Neumann boundary conditions

We give a more detailed account of Theorem 2.3 in the particular case of Neumann boundary conditions

2.5a $$\begin{aligned} \delta =1\,, \end{aligned}$$

so that $\mu _0=0$, and spatially homogeneous data

2.5b $$\begin{aligned}&d\in C(J)\,,\quad d(a)\ge {\underline{d}}>0\,,\quad a\in J\,, \end{aligned}$$

2.5c $$\begin{aligned}&m\in C(J)\,,\quad m\ge 0\,, \qquad b\in L_\infty (J)\,,\quad b> 0\,, \end{aligned}$$

that is, data only depending on age *a*. In this particular situation, besides the trivial steady state $(S_*,I_*)=(0,0)$ and the disease-free steady state $(S_*,I_*)=(\kappa _2,0)$, there is also an *endemic steady state* $({{\bar{S}}}_*,{{\bar{I}}}_*)$ provided that ${\textsf{R}}_0>1$, where

2.6 $$\begin{aligned} {\textsf{R}}_0 :=\kappa _2\int _0^{a_m} b(a) \Pi (a)\, \textrm{d}a \end{aligned}$$

with

$$\begin{aligned} \Pi (a):=\exp \left( -\int _0^a m(\sigma )\,\textrm{d}\sigma \right) ,\quad a\in J. \end{aligned}$$

It is given as

$$\begin{aligned} {{\bar{S}}}_*:=\frac{\kappa _2}{{\textsf{R}}_0 },\qquad {{\bar{I}}}_*(a):= \frac{1}{{\textsf{R}}_0 } \kappa _1\kappa _2\left( 1-\frac{1}{{\textsf{R}}_0 }\right) \Pi (a),\quad a\in J. \end{aligned}$$

Linear stability and instability of these steady states is determined by the basic reproduction number ${\textsf{R}}_0$:

#### Theorem 2.4

Assume (2.4), (2.5), let $p>(2\vee n)$, and let ${\textsf{R}}_0>0$ be defined in (2.6).

$$\mathbf{(a)}$$
The trivial steady state $(S_*,I_*)=(0,0)$ is linearly unstable in the space $L_p(\Omega )\times L_1(J,L_p(\Omega ))$.
$$\mathbf{(b)}$$
If ${\textsf{R}}_0 <1$, then the disease-free steady state $(S_*,I_*)=(\kappa _2,0)$ is globally linearly stable in the space $L_p(\Omega )\times L_1(J,L_p(\Omega ))$; that is, it is linearly stable and attracts any solution starting from positive initial values. If ${\textsf{R}}_0 >1$, then $(S_*,I_*)=(\kappa _2,0)$ is linearly unstable in $L_p(\Omega )\times L_1(J,L_p(\Omega ))$.
$$\mathbf{(c)}$$
For $1<{\textsf{R}}_0 <3$, the endemic steady state $({{\bar{S}}}_*,{{\bar{I}}}_*)$ is linearly stable in the space $L_p(\Omega )\times L_1(J,L_p(\Omega ))$.

Part (a) and the local stability statements of part (b) of Theorem 2.4 have been observed already in Theorem 2.3. The proofs of the remaining statements of Theorem 2.4 are given in Sect. 6. In fact, when ${\textsf{R}}_0 <1$ we prove that

$$\begin{aligned} \lim _{t\rightarrow \infty } \big (S(t),I(t)\big )=(\kappa _2,0)\ \text { in }\ L_p(\Omega )\times L_1(J, C({{\bar{\Omega }}})) \end{aligned}$$

for any solution (*S*, *I*) to (1.1) corresponding to positive nontrivial initial values $(S_0,I_0)$ so that there is no further steady state in this case (in accordance with Theorem 2.3 (c)). Clearly, one expects $({{\bar{S}}}_*,{{\bar{I}}}_*)$ to be linearly stable whenever ${\textsf{R}}_0 >1$.

## Well-posedness: Proof of Theorem 2.1

We prove Theorem 2.1 in several steps. After introducing some notation we derive the existence of a local solution and then establish further properties.

### Preliminaries and notation

For two Banach spaces *E* and *F* we write $\mathcal {L}(E,F)$ for the Banach space of bounded linear operators from *E* to *F*, and we set $\mathcal {L}(E):=\mathcal {L}(E,E)$. Similarly, $\mathcal {K}(E,F)$ and $\mathcal {K}(E)$ stand for compact linear operators.

For fixed $\delta \in \{0,1\}$ and $p\in (1,\infty )$, we set

$$\begin{aligned} \mathcal {B}u:=u \ \text { on } \ \partial \Omega \ \text { if } \ \delta =0,\qquad \mathcal {B}u:=\partial _\nu u\ \text { on } \ \partial \Omega \ \text { if } \ \delta =1, \end{aligned}$$

and introduce the scale of Banach spaces

3.1 $$\begin{aligned} W_{p,\mathcal {B}}^{2\theta }(\Omega ):=\left\{ \begin{array}{ll} \{v\in W_{p}^{2\theta }(\Omega );\; \mathcal {B} w=0 \text { on } \partial \Omega \}, &{} \delta +\frac{1}{p}<2\theta \le 2,\\ W_{p}^{2\theta }(\Omega ), &{} 0\le 2\theta <\delta +\frac{1}{p}.\end{array} \right. \end{aligned}$$

By $\Delta _\mathcal {B}$ we denote the Laplacian defined on $W_{p,\mathcal {B}}^2(\Omega )$. Moreover, for fixed $a\in J$, also the operator

3.2 $$\begin{aligned} A(a):=d(a)\Delta _\mathcal {B} -m(a,\cdot )-r(a,\cdot ) \end{aligned}$$

has domain $W_{p,\mathcal {B}}^2(\Omega )$. Then $\Delta _\mathcal {B}$ and *A*(*a*) are generators of positive analytic contraction semigroups on $L_p(\Omega )$ for each $p\in (1,\infty )$, see Amann (1983); Rothe (1984). In fact, since

$$\begin{aligned} A\in C^\rho \big (J,\mathcal {L}(W_{p,\mathcal {B}}^2(\Omega ),L_p(\Omega ))\big ), \end{aligned}$$

it follows from Amann (1995, II.Corollary 4.4.2) that *A* generates a positive parabolic evolution operator

$$\begin{aligned} U_A(a,\sigma ),\quad 0\le \sigma \le a\le a_m, \end{aligned}$$

on $L_p(\Omega )$ in the sense of Amann (1995, II.Section 2.1). In particular,

$$\begin{aligned} v(a):=U_{A}(a,\sigma )v^0,\quad a\in [\sigma ,a_m], \end{aligned}$$

is, for given $\sigma \in [0,a_m)$ and $v^0\in L_p(\Omega )$, the unique solution

$$\begin{aligned} v\in C\big ([\sigma ,a_m],L_p(\Omega )\big ) \cap C^1\big ((\sigma ,a_m],L_p(\Omega )\big )\cap C\big ((\sigma ,a_m),W_{p,\mathcal {B}}^2(\Omega )\big ) \end{aligned}$$

to the Cauchy problem

$$\begin{aligned} \partial _a v(a)=A(a) v(a),\quad a\in (\sigma ,a_m),\qquad v(\sigma )=v^0. \end{aligned}$$

The contraction properties

3.3 $$\begin{aligned} \begin{aligned} \Vert e^{t\Delta _\mathcal {B}}\Vert _{\mathcal {L}(L_p(\Omega ))}&\le 1,\quad t\ge 0,\\ \Vert U_A(a,\sigma )\Vert _{\mathcal {L}(L_p(\Omega ))}&\le e^{-\int _\sigma ^a{\underline{m}}(r)\,\textrm{d}r}\,,\quad 0\le \sigma \le a\in J, \end{aligned} \end{aligned}$$

are valid, where

$$\begin{aligned} {\underline{m}}(a):=\mathop {\mathrm {ess-inf}}\limits _{x\in \Omega } m(a,x)\ge 0,\quad a\in J. \end{aligned}$$

Recall the interpolation relations

$$\begin{aligned} \big (L_p(\Omega ),W_{p,\mathcal {B}}^2(\Omega )\big )_{\theta ,p}\doteq W_{p,\mathcal {B}}^{2\theta }(\Omega ),\quad 2\theta \in [0,2]\setminus \left\{ 1,\delta +\frac{1}{p}\right\} , \end{aligned}$$

with real interpolation functor $(\cdot ,\cdot )_{\theta ,p}$ and

$$\begin{aligned} \big [L_p(\Omega ),W_{p,\mathcal {B}}^2(\Omega )\big ]_{1/2}\doteq W_{p,\mathcal {B}}^{1}(\Omega ) \end{aligned}$$

with complex interpolation functor $[\cdot ,\cdot ]_{1/2}$ (see Triebel (1978, 4.4.3/Theorem)). From Amann (1995, II. Lemma 5.1.3) and the embedding $W_{p,\mathcal {B}}^{2\theta }(\Omega )\hookrightarrow L_q(\Omega )$ for $\theta =\frac{n}{2}(\frac{1}{p}-\frac{1}{q})$ we then infer parabolic regularizing properties in the sense that, given $0\le \vartheta \le \theta \le 1$ with $2\vartheta , 2\theta \notin \{\delta +\frac{1}{p}\}$ and $1<p\le q\le \infty$, there are $\varpi \in \mathbb {R}$ and $M\ge 1$ such that

3.4 $$\begin{aligned} t^{\theta -\vartheta }\,\Vert e^{t\Delta _\mathcal {B}}\Vert _{\mathcal {L}(W_{p,\mathcal {B}}^{2\vartheta }(\Omega ),W_{p,\mathcal {B}}^{2\theta }(\Omega ))}+ t^{\frac{n}{2}(\frac{1}{p}-\frac{1}{q})}\,\Vert e^{t\Delta _\mathcal {B}}\Vert _{\mathcal {L}(L_p(\Omega ),L_q(\Omega ))} \le M \, e^{\varpi t}\,,\quad t>0\,, \end{aligned}$$

and

3.5 $$\begin{aligned}&(a-\sigma )^{\theta -\vartheta }\, \Vert U_A(a,\sigma )\Vert _{\mathcal {L}(W_{p,\mathcal {B}}^{2\vartheta }(\Omega ),W_{p,\mathcal {B}}^{2\theta }(\Omega ))}+ (a-\sigma )^{\frac{n}{2}(\frac{1}{p}-\frac{1}{q})}\, \Vert U_A(a,\sigma )\Vert _{\mathcal {L}(L_p(\Omega ),L_q(\Omega ))}\nonumber \\&\qquad \qquad \le M \, e^{\varpi (a-\sigma )}\,. \end{aligned}$$

Let $p\in \big (\max \{\frac{3n}{4},2\},\infty \big )$ and let $S_0\in L_p(\Omega )$ and $I_0\in L_1(J,L_p(\Omega ))$ be fixed in the following.

### Existence of a unique maximal solution

Given $S\in L_p(\Omega )$ and $I\in L_1(J,L_p(\Omega ))$ we use the abbreviations (dropping *x*-dependence for simplicity)

$$\begin{aligned} B[S,I]:=S\int _0^{a_m} b(a)\,I(a)\,\textrm{d}a,\qquad R[I]:=\int _0^{a_m} r(a)\,I(a)\,\textrm{d}a, \end{aligned}$$

and

$$\begin{aligned} f[S,I]:=\kappa _1\left( 1-\frac{S}{\kappa _2}\right) S-B[S,I]+R[I], \end{aligned}$$

where as, for time-dependent functions

$$\begin{aligned}S:[0,T]\rightarrow L_p(\Omega ) \quad \hbox {and}\quad I:[0,T]\rightarrow L_1(J,L_p(\Omega )), \end{aligned}$$

it is convenient to abbreviate

$$\begin{aligned} B[S,I](t):=B[S(t),I(t)],\quad f[S,I](t):=f[S(t),I(t)],\qquad t\in [0,T]. \end{aligned}$$

Then (1.1) can be written compactly as

3.6a $$\begin{aligned} \partial _t S(t)&=\Delta _\mathcal {B} S(t) +f[S,I](t)\,,\qquad t>0\,, \end{aligned}$$

3.6b $$\begin{aligned} D I(t,a)&=A(a)I(t,a)\,,\qquad t>0\,,\quad a\in J\,, \end{aligned}$$

3.6c $$\begin{aligned} I(t,0)&=B[S,I](t)\,,\qquad t>0 \,, \end{aligned}$$

subject to the initial conditions

3.6d $$\begin{aligned} S(0)=S_0\,,\qquad I(0,a)&= I_0(a)\,,\quad a\in J\,. \end{aligned}$$

Solutions *S* to (3.6a) are of the form

$$\begin{aligned} \mathcal {S}[S,I](t):= e^{t\Delta _\mathcal {B}}S_0+\displaystyle \int _0^t e^{(t-\tau )\Delta _\mathcal {B}}f[S,I](\tau )\,\textrm{d}\tau ,\quad t>0, \end{aligned}$$

while integrating (3.6b) subject to (3.6c) formally along characteristics (and recalling the properties of the evolution operator $U_A$) yields a solution *I* in the form

$$\begin{aligned} \mathcal {I}[S,I](t,a):=\left\{ \begin{array}{ll} U_A(a,a-t) I_0(a-t),&{} a>t, \ a\in J,\\ U_A(a,0)B[S,I](t-a) ,&{}a\le t,\ a\in J. \end{array} \right. \end{aligned}$$

Given $T>0$ we introduce the Banach space

$$\begin{aligned} \mathbb {X}_T:=C\big ([0,T],L_p(\Omega )\times L_1(J,L_p(\Omega ))\big ) \end{aligned}$$

and define

$$\begin{aligned} \mathcal {Y}[S,I](t):=\big ( \mathcal {S}[S,I](t) ,\, \mathcal {I}[S,I](t,\cdot )\big ),\qquad t\in [0,T],\quad (S,I)\in \mathbb {X}_T. \end{aligned}$$

Then, fixed points (*S*, *I*) of $\mathcal {Y}$ correspond to solutions of (3.6). In order to prove that $\mathcal {Y}$ has a fixed point we first note:

#### Lemma 3.1

For $q=p/2$, the mappings

3.7 $$\begin{aligned}{} & {} B:L_p(\Omega )\times L_1\big (J,L_p(\Omega )\big ) \rightarrow L_{q}(\Omega ), \nonumber \\{} & {} f:L_p(\Omega )\times L_1\big (J,L_p(\Omega )\big ) \rightarrow L_{q}(\Omega ) \end{aligned}$$

are uniformly Lipschitz continuous on bounded sets. Moreover, if $2\theta >n/p$, then there is $\alpha >0$ such that

$$\begin{aligned}{} & {} B:W_p^{2\theta }(\Omega )\times L_1\big (J,W_p^{2\theta }(\Omega )\big ) \rightarrow W_p^{2\alpha }(\Omega ),\nonumber \\{} & {} f:W_p^{2\theta }(\Omega )\times L_1\big (J,W_p^{2\theta }(\Omega )\big ) \rightarrow W_p^{2\alpha }(\Omega ) \end{aligned}$$

are uniformly Lipschitz continuous on bounded sets.

#### Proof

The statements readily follow from the regularity assumptions (2.1) and the fact that pointwise multiplications

3.8 $$\begin{aligned} L_p(\Omega )\times L_p(\Omega )\rightarrow L_{p/2}(\Omega )\quad \text { and }\quad W_p^{2\theta }(\Omega )\times W_p^{2\theta }(\Omega )\rightarrow W_p^{2\alpha }(\Omega ) \end{aligned}$$

are continuous for some $\alpha >0$, see Amann (1991, Theorem 4.1). $\square$

#### Proposition 3.2

Given $R>0$ there is $T=T(R)>0$ such that, if

$$\begin{aligned} \Vert S_0\Vert _{L_p(\Omega )}+\Vert I_0\Vert _{L_1(J,L_p(\Omega ))}<R, \end{aligned}$$

then

$$\begin{aligned} \mathcal {Y}:{{\bar{\mathbb {B}}}}_{\mathbb {X}_T}(0,R)\rightarrow {{\bar{\mathbb {B}}}}_{\mathbb {X}_T}(0,R) \end{aligned}$$

has a unique fixed point (*S*, *I*).

#### Proof

Let $T\in (0,1)$ and $\Vert S_0\Vert _{L_p(\Omega )}+\Vert I_0\Vert _{L_1(J,L_p(\Omega ))}<R$. Considering $(S,I), ({\tilde{S}},{\tilde{I}})\in {\mathbb {X}_T}$ both with norm less than *R*, we have

$$\begin{aligned} f[S,I]\in C\big ([0,T],L_{p/2}(\Omega )\big ) \end{aligned}$$

by Lemma 3.1 so that we readily obtain that $\mathcal {S}[S,I]\in C\big ([0,T],L_{p}(\Omega )\big )$ by (3.4) since $t\mapsto t^{-n/2p}$ is integrable on (0, *T*) as $2p>n$. Moreover,

3.9 $$\begin{aligned} \Vert \mathcal {S}[S,I](t)\Vert _{L_{p}(\Omega )}\le \Vert S_0\Vert _{L_p(\Omega )}+c(R)\, T^{1-n/2p} \,,\quad t\in [0,T]\,, \end{aligned}$$

and

3.10 $$\begin{aligned} \Vert \mathcal {S}[S,I](t)-\mathcal {S}[{\tilde{S}}, \tilde{I}](t)\Vert _{L_{p}(\Omega )}\le c(R)\, T^{1-n/2p}\,\Vert (S,I)-(\tilde{S},{\tilde{I}})\Vert _{\mathbb {X}_T}\,,\quad t\in [0,T]\,. \end{aligned}$$

From (3.3)–(3.5) and Lemma 3.1 we infer^1^

3.11 $$\begin{aligned} ||\mathcal {I}[S,I](t)\Vert _{L_1(J,L_p(\Omega ))}&\le \int _0^t\Vert U_A(a,0)\Vert _{\mathcal {L}(L_{p/2}(\Omega ),L_p(\Omega ))}\,\Vert B[S,I](t-a)\Vert _{L_{p/2}(\Omega )}\,\textrm{d}a\nonumber \\&\qquad + \int _t^{a_m}\Vert U_A(a,a-t)\Vert _{\mathcal {L}(L_p(\Omega ))}\,\Vert I_0(a-t)]\Vert _{L_p(\Omega )}\,\textrm{d}a\nonumber \\&\le c(R) T^{1-n/2p}+\Vert I_0\Vert _{L_1(J,L_p(\Omega ))} \end{aligned}$$

for $t\in [0,T]$, and similarly

3.12 $$\begin{aligned}&\Vert \mathcal {I}[S,I](t)-\mathcal {I}[{\tilde{S}}, {\tilde{I}}](t)\Vert _{L_1(J, L_{p}(\Omega ))}\nonumber \\&\qquad \le \int _0^t\Vert U_A(a,0)\Vert _{\mathcal {L}(L_{p/2}(\Omega ),L_p(\Omega ))}\,\Vert B[S,I](t-a)-B[{\tilde{S}},{\tilde{I}}](t-a)\Vert _{L_{p/2}(\Omega )}\,\textrm{d}a \nonumber \\&\qquad \le c(R)\, T^{1-n/2p}\,\Vert (S,I)-({\tilde{S}},{\tilde{I}})\Vert _{\mathbb {X}_T}\,. \end{aligned}$$

To check continuity we use (3.3)–(3.5) together with Lemma 3.1 and write, for $0\le t_2\le t_1\le T$,

$$\begin{aligned} ||\mathcal {I}[S,I](t_1)&-\mathcal {I}[S,I](t_2)\Vert _{L_1(J,L_p(\Omega ))}\\&\le \int _0^{t_2}\Vert U_A(a,0)\Vert _{\mathcal {L}(L_{p/2}(\Omega ),L_p(\Omega ))}~\Vert B[S,I](t_1-a)\!-\!B[S,I](t_2-a)\Vert _{L_{p/2}(\Omega )}~\textrm{d}a\\&\quad +\int _{t_2}^{t_1}\Vert U_A(a,0)\Vert _{\mathcal {L}(L_{p/2}(\Omega ),L_p(\Omega ))}\,\Vert B[S,I](t_1-a)\Vert _{L_{p/2}(\Omega )}\,\textrm{d}a\\&\quad + \int _{t_2}^{t_1}\Vert U_A(a,a-t_2)\Vert _{\mathcal {L}(L_{p}(\Omega ))}\,\Vert I_0(a-t_2)\Vert _{L_p(\Omega )}\,\textrm{d}a\\&\quad + \int _{t_1}^{a_m}\big \Vert \big (U_A(a,a-t_1)-U_A(a,a-t_2)\big ) I_0(a-t_1)\Vert _{L_p(\Omega )}\,\textrm{d}a\\&\quad + \int _{t_1}^{a_m} \Vert U_A(a,a-t_2)\Vert _{\mathcal {L}(L_p(\Omega ))}\,\Vert I_0(a-t_1)-I_0(a-t_2)\Vert _{L_p(\Omega )}\,\textrm{d}a\\&\le Me^\varpi \int _0^{t_2} a^{-n/2p}\,\Vert B[S,I](t_1-a)-B[S,I](t_2-a)\Vert _{L_{p/2}(\Omega )}\,\textrm{d}a\\&\quad +c(R) \int _{t_2}^{t_1}a^{-n/2p}\,\textrm{d}a + \int _{t_2}^{t_1}\Vert I_0(a-t_2)\Vert _{L_p(\Omega )}\,\textrm{d}a\\&\quad + \int _{t_1}^{a_m}\big \Vert \big (U_A(a,a-t_1)-U_A(a,a-t_2)\big ) I_0(a-t_1)\Vert _{L_p(\Omega )}\,\textrm{d}a\\&\quad + \int _{t_1}^{a_m} \Vert I_0(a-t_1)-I_0(a-t_2)\Vert _{L_p(\Omega )}\,\textrm{d}a\,. \end{aligned}$$

Now, as $\vert t_1-t_2\vert \rightarrow 0$, the first integral on the right-hand side goes to zero since the function $B[S,I]\in C\big ([0,T],L_{p/2}(\Omega )\big )$ is uniformly continuous while the second and the third integral vanish since $a\mapsto a^{-n/2p}$ respectively $I_0$ are integrable. To see that the fourth integral vanishes in the limit one may use the strong continuity of the evolution operator $U_A$ on $L_p(\Omega )$ (Amann 1995, Equation II. (2.1.2)) and Lebesgue’s theorem. Finally, for the last integral one may use the strong continuity of the translations on $L_1(J,L_p(\Omega ))$. Consequently, $\mathcal {I}[S,I]\in C\big ([0,T],L_1(J,L_p(\Omega ))\big )$.

Summarizing, we have shown in (3.9)-(3.12) that, given

$$\begin{aligned} \Vert S_0\Vert _{L_p(\Omega )}+\Vert I_0\Vert _{L_1(J,L_p(\Omega ))}<R, \end{aligned}$$

we can choose $T=T(R)\in (0,1)$ such that

$$\begin{aligned} \mathcal {Y}:{{\bar{\mathbb {B}}}}_{\mathbb {X}_T}(0,R)\rightarrow {{\bar{\mathbb {B}}}}_{\mathbb {X}_T}(0,R) \end{aligned}$$

is a contraction, and the claim follows from Banach’s fixed point theorem. $\square$

Since $T=T(R)$ in the proof of Proposition 3.2 depends only upon

$$\begin{aligned} R>\Vert S_0\Vert _{L_p(\Omega )}+\Vert I_0\Vert _{L_1(J,L_p(\Omega ))}, \end{aligned}$$

it is standard to extend (*S*, *I*) to a maximal solution and to show that the solution map defines a semiflow:

#### Corollary 3.3

(*S*, *I*) can be extended to a maximal interval $$[0,T_m)$$ such that

$$\begin{aligned} (S,I)\in C\big ([0,T_m),L_p(\Omega )\times L_1(J,L_p(\Omega ))\big ) \end{aligned}$$

satisfies

3.13 $$\begin{aligned} S(t)= e^{t\Delta _\mathcal {B}}S_0+\displaystyle \int _0^t e^{(t-\tau )\Delta _\mathcal {B}}f[S,I](\tau )\,\textrm{d}\tau ,\quad t\in [0,T_m), \end{aligned}$$

and

3.14 $$\begin{aligned} I(t,a)=\left\{ \begin{array}{ll} U_A(a,a-t) I_0(a-t),&{} a\ge t, \ \quad (a,t)\in J\times [0,T_m),\\ U_A(a,0)B[S,I](t-a) ,&{}a< t,\ \quad (a,t)\in J\times [0,T_m). \end{array} \right. \end{aligned}$$

If $$T_m<\infty $$, then

3.15 $$\begin{aligned} \lim _{t\nearrow T_m}\big (\Vert S(t)\Vert _{L_p(\Omega )}+\Vert I(t,\cdot )\Vert _{L_1(J,L_p(\Omega ))}\big )=\infty . \end{aligned}$$

Moreover, the mapping $\big (t,(S_0,I_0)\big )\mapsto (S,I)(t)$ defines a semiflow on the space $L_p(\Omega )\times L_1(J,L_p(\Omega ))$.

#### Remark 3.4

It is worth noting that Corollary 3.3 remains valid for models that can be recast in the form (3.6) such that *f* and *B* satisfy (3.7) with $\frac{n}{2}(\frac{1}{q}-\frac{1}{p})<1$ for some $1<q\le p<\infty$.

### Regularity

We derive further regularity properties of the solution (*S*, *I*) (it is for this step that we have imposed restrictive regularity assumptions on the data *b* and *r*).

#### Proposition 3.5

Let $2\vartheta \in [0,2]\setminus \{\delta +\frac{1}{p}\}$. If $S_0\in W_{p,\mathcal {B}}^{2\vartheta }(\Omega )$, then

$$\begin{aligned} S\in C^1\big ((0, T_m),L_p(\Omega )\big )\cap C\big ((0, T_m),W_{p,\mathcal {B}}^{2}(\Omega )\big )\cap C\big ([0, T_m),W_{p,\mathcal {B}}^{2\vartheta }(\Omega )\big ) \end{aligned}$$

is a strong solution to (1.1a) while, if $I_0\in L_1(J,W_{p,\mathcal {B}}^{2\vartheta }(\Omega ))$, then

$$\begin{aligned} I\in C\big ((0,T_m),L_1(J,W_{p,\mathcal {B}}^{2}(\Omega ))\big )\cap C\big ([0, T_m),L_1(J,W_{p,\mathcal {B}}^{2\vartheta }(\Omega )))\big ) \end{aligned}$$

satisfies (1.1b) in the sense that

$$\begin{aligned} D I(t,a)=A(a)I(t,a)\ \text { in }\ L_p(\Omega ) \end{aligned}$$

for $$t\in (0,T_m)$$ and a.e. $$a\in (0,a_m)$$.

#### Proof

Since

$$\begin{aligned} \Arrowvert e^{t\Delta _\mathcal {B}}\Arrowvert _{\mathcal {L}(L_{p/2},W_{p,\mathcal {B}}^{2\theta }(\Omega ))}\le c(T)t^{-n/2p-\theta },\quad 0< t\le T, \end{aligned}$$

and $f[S,I]\in C\big ([0,T_m),L_{p/2}(\Omega )\big )$, it readily follows from (3.13) that $S\in C\big ((0,T_m),W_{p,\mathcal {B}}^{2\theta }(\Omega )\big )$ for $2\theta <2-n/p$ with $2\theta \notin \{\delta +1/p\}$. Similarly, as in the proof of Proposition 3.2 (see also the proof of Lemma 7.1 in the Appendix) one derives from

$$\begin{aligned} \Vert U_A(a,\sigma )\Vert _{\mathcal {L}(L_{p/2}(\Omega ),W_{p,\mathcal {B}}^{2\theta }(\Omega ))}\le M (a-\sigma )^{-n/2p-\theta }\,,\quad 0\le \sigma \le a\in J\,, \end{aligned}$$

and $B[S,I]\in C\big ([0,T_m),L_1(J,L_{p/2}(\Omega ))\big )$ that $I\in C\big ((0,T_m),L_1(J,W_{p,\mathcal {B}}^{2\theta }(\Omega ))\big )$ for $2\theta <2-n/p$ with $2\theta \notin \{\delta +1/p\}$. Now, since $n<4p/3$, we find some $2\theta \in (n/2p,2-n/p)\setminus \{\delta +1/p\}$ so that, according to Lemma 3.1, there is $\alpha >0$ such that

$$\begin{aligned} f[S,I](\varepsilon +\cdot )\in C\big ([0, T_m-\varepsilon ),W_{p,\mathcal {B}}^{2\alpha }(\Omega )\big ) \end{aligned}$$

for each $\varepsilon >0$ small. Thus, we infer from Amann (1995, II.Theorem 1.2.2) that

$$\begin{aligned} S_\varepsilon :=S(\varepsilon +\cdot )\in C^1\big ((0, T_m-\varepsilon ),L_p(\Omega )\big )\cap C\big ((0, T_m-\varepsilon ),W_{p,\mathcal {B}}^{2}(\Omega )\big ) \end{aligned}$$

is a strong solution to

$$\begin{aligned} \partial _t S_\varepsilon =\Delta _\mathcal {B} S_\varepsilon +f[S,I](\varepsilon +\cdot ),\quad t\in (0, T_m-\varepsilon ),\qquad S_\varepsilon (0)=S(\varepsilon )\in W_{p,\mathcal {B}}^{2\theta }(\Omega ). \end{aligned}$$

Letting then $\varepsilon$ tend to zero we obtain that

$$\begin{aligned} S\in C^1((0, T_m),L_p(\Omega ))\cap C((0, T_m),W_{p,\mathcal {B}}^{2}(\Omega )) \end{aligned}$$

is a strong solution to (1.1a). Moreover, if $S_0\in W_{p,\mathcal {B}}^{2\vartheta }(\Omega )$ for some $2\vartheta \in [0,2]{\setminus }\{\delta +1/p\}$, then

$$\begin{aligned} S\in C\big ([0, T_m),W_{p,\mathcal {B}}^{2\vartheta }(\Omega )\big ). \end{aligned}$$

Similarly, setting

$$\begin{aligned} I_\varepsilon :=I(\varepsilon +\cdot ,\cdot ),\qquad I_{0,\varepsilon }:=I_\varepsilon (0,\cdot )=I(\varepsilon ,\cdot ), \end{aligned}$$

we deduce from (3.14) and the properties of evolution operators that, for $t\in [0,T_m-\varepsilon )$ and $a\in J$,

$$\begin{aligned} I_\varepsilon (t,a)&=\left\{ \begin{array}{ll} U_A(a,a-t-\varepsilon ) I_0(a-t-\varepsilon )\,,&{} a>t+\varepsilon \,, \\ U_A(a,0)B[S,I](\varepsilon +t-a) \,,&{}a\le t+\varepsilon \,, \end{array} \right. \\&=\left\{ \begin{array}{ll} U_A(a,a-t)U_A(a-t,a-t-\varepsilon ) I_0(a-t-\varepsilon )\,,&{} a>t+\varepsilon \,,\\ U_A(a,a-t)U_A(a-t,0)B[S,I](\varepsilon +t-a) \,,&{}t<a\le t+\varepsilon \,, \\ U_A(a,0)B[S,I](\varepsilon +t-a) \,,&{}a\le t\,, \end{array} \right. \\&=\left\{ \begin{array}{ll} U_A(a,a-t) I_{0,\varepsilon }(a-t)\,,&{} a>t\,, \\ U_A(a,0)B[S_\varepsilon ,I_\varepsilon ](t-a) \,,&{}a\le t\,. \end{array} \right. \end{aligned}$$

Now, since

$$\begin{aligned}{} & {} S_\varepsilon \in C\big ([0, T_m-\varepsilon ),W_{p,\mathcal {B}}^{2}(\Omega )\big ),\quad I_\varepsilon \in C\big ([0, T_m-\varepsilon ),L_1(J,W_{p,\mathcal {B}}^{2\theta }(\Omega ))\big ), \\{} & {} \qquad I_{0,\varepsilon }\in L_1(J,W_{p,\mathcal {B}}^{2\theta }(\Omega )) \end{aligned}$$

for $2\theta <2-n/p$, it follows from (3.5) (see Lemma 7.1 in the Appendix) that

$$\begin{aligned} I\in C\big ((0,T_m),L_1(J,W_{p,\mathcal {B}}^{2}(\Omega ))\big ). \end{aligned}$$

In addition, if $I_0\in L_1(J,W_{p,\mathcal {B}}^{2\vartheta }(\Omega ))$ for some $2\vartheta \in [0,2]\setminus \{\delta +1/p\}$, then

$$\begin{aligned} I\in C\big ([0, T_m),L_1(J,W_{p,\mathcal {B}}^{2\vartheta }(\Omega ))\big ). \end{aligned}$$

Moreover, (3.14) and the differentiability properties of the evolution operator $U_A$ stated in Amann (1995, II.Equation (2.1.6)) imply

$$\begin{aligned} D I(t,a)=\lim _{h\rightarrow 0^+} \frac{1}{h}\big (I(t+h,a+h)-I(t,a)\big )=A(a)I(t,a)\ \text { in }\ L_p(\Omega ) \end{aligned}$$

for $t\in (0,T_m)$ and a.e. $a\in (0,a_m)$ (in fact, for every $a\in (0,a_m)$ provided $I_0\in C\big ((0,a_m), L_p(\Omega )\big )$ is continuous). $\square$

Note that taking $2\vartheta =0$ in Proposition 3.5 we obtain the regularity of the solution (*S*, *I*) claimed in Theorem 2.1.

#### Remark 3.6

Assuming additionally $b\in BC^1(J,C({{\bar{\Omega }}}))$ and $I_0\in C^1(J,L_p(\Omega ))$, one can show analogously to Walker (2010, Proposition 1) that the partial derivatives $\partial _t I(t,a)$ and $\partial _a I(t,a)$ exist and

$$\begin{aligned} D I(t,a)= \partial _t I(t,a)+ \partial _a I(t,a)=A(a) I(t,a) \end{aligned}$$

in $L_p(\Omega )$ for $t\in (0,T_m)$ and $a\in (0,a_m)$.

### Positivity

Since the semigroup $\big (e^{t\Delta _\mathcal {B}}\big )_{t\ge 0}$ and the evolution operator $\big (U_A(a,\sigma )\big )_{0\le \sigma \le a\le a_m}$ are positive operators on $L_p(\Omega )$ (as well as on the spaces $W_{p,\mathcal {B}}^{2\theta }(\Omega )$) and since there is $\omega (R)>0$ such that

$$\begin{aligned} B[S,I]\ge 0,\quad f[S,I]+\omega (R)S\ge 0 \end{aligned}$$

provided that $S,I\ge 0$ with $\Vert (S,I)\Vert _{L_\infty (\Omega )\times L_1(J,L_\infty (\Omega ))}\le R$ (see Sect. 3.3 for such local bounds), it is a standard iteration argument to derive that the solution (*S*, *I*) from Corollary 3.3 corresponding to non-negative initial values $S_0\in L_p^+(\Omega )$ and $I_0\in L_1(J,L_p^+(\Omega ))$ satisfies ${S}(t)\in {L}_{p}^+(\Omega )$ and ${{I}}({{t}})\in {{L}}_1({{J,L}}_{{p}}^+(\Omega ))$ for $t\in [0,T_m)$.

### Global existence

Integrating (1.1) yields for $t\in (0,T_m)$ the inequality (in fact, equality for Neumann boundary conditions, see Sect. 1 in the Appendix for a rigorous proof)

3.16 $$\begin{aligned} \int _\Omega S(t,x)\,&\textrm{d}x+\int _0^{a_m}\int _\Omega I(t,a,x)\,\textrm{d}x\,\textrm{d}a\nonumber \\ \le&\int _\Omega S_0(x)\,\textrm{d}x+\int _0^{a_m}\int _\Omega I_0(a,x)\,\textrm{d}x\,\textrm{d}a+\int _0^t\int _\Omega \kappa _1\left( 1-\frac{S(\tau ,x)}{\kappa _2}\right) S(\tau ,x)\,\textrm{d}x\,\textrm{d}\tau \nonumber \\&-\int _0^t\int _0^{a_m}\int _\Omega m(a,x)\,I(\tau ,a,x)\,\textrm{d}x\,\textrm{d}a\,\textrm{d}\tau -\int _{a_m-t}^{a_m}\int _\Omega I_0(a,x)\,\textrm{d}x\,\textrm{d}a\nonumber \\&+\int _0^t\int _{a_m-t+\tau }^{a_m}\int _\Omega \big (m(a,x)+r(a,x)\big )\,U_A(a,a-\tau )\,I_0(a-\tau ,x)\,\textrm{d}x\,\textrm{d}a\,\textrm{d}\tau \,. \end{aligned}$$

Since

3.17 $$\begin{aligned} \kappa _1\left( 1-\frac{S}{\kappa _2}\right) S\le \frac{\kappa _1\kappa _2}{4}, \end{aligned}$$

we thus deduce from the positivity of (*S*, *I*) the $L_1$-estimate

3.18 $$\begin{aligned} \Vert S(t)\Vert _{L_1(\Omega )}+\Vert I(t)\Vert _{L_1(J,L_1(\Omega ))}&\le \, \Vert S_0\Vert _{L_1(\Omega )}+\Vert I_0\Vert _{L_1(J,L_1(\Omega ))} \nonumber \\&\quad +t\vert \Omega \vert \frac{\kappa _1\kappa _2}{4}+\Vert m+r||_{L_\infty (J,L_\infty (\Omega ))} t\Vert I_0\Vert _{L_1(J,L_1(\Omega ))} \nonumber \\ \end{aligned}$$

for $t\in (0,T_m)$. We shall then proceed with the following auxiliary result:

#### Lemma 3.7

**(i)** Let $1\le q\le r\le \infty$ with $\frac{n}{2}(\frac{1}{q}-\frac{1}{r})<1$. If

$$\begin{aligned} \Vert I(t)\Vert _{L_1(J,L_q(\Omega ))}\le c_0(T),\quad t\in [0,T], \end{aligned}$$

then

$$\begin{aligned} \Vert S(t)\Vert _{L_r(\Omega )}\le \Vert S_0\Vert _{L_r(\Omega )}+c(T),\quad t\in [0,T]. \end{aligned}$$

**(ii)** Let $1\le r\le \infty$ with $\frac{n}{2r}<1$. If

$$\begin{aligned} \Vert S(t)\Vert _{L_r(\Omega )}\le c_0(T),\quad t\in [0,T], \end{aligned}$$

then

$$\begin{aligned} \Vert I(t)\Vert _{L_1(J,L_p(\Omega ))}\le c(T)\big (1+\Vert I_0\Vert _{L_1(J,L_p(\Omega ))}\big ) ,\quad t\in [0,T]. \end{aligned}$$

#### Proof

**(i)** By (3.13) we have

$$\begin{aligned} 0\le S(t)\le \,&e^{t\Delta _\mathcal {B}}S_0+\displaystyle \int _0^t e^{(t-\tau )\Delta _\mathcal {B}}\kappa _1\left( 1-\frac{S(\tau )}{\kappa _2}\right) S(\tau )\,\textrm{d}\tau \\&+\displaystyle \int _0^t e^{(t-\tau )\Delta _\mathcal {B}}\int _0^{a_m} r(a,\cdot )I(\tau ,a)\,\textrm{d}a\,\textrm{d}\tau \end{aligned}$$

and therefore, using (3.17) and

$$\begin{aligned} \Vert e^{(t-\tau )\Delta _\mathcal {B}}\Vert _{\mathcal {L}(L_q(\Omega ),L_r(\Omega ))}\le c(T) (t-\tau )^{-\frac{n}{2}(\frac{1}{q}-\frac{1}{r})},\quad 0\le \tau <t\le T, \end{aligned}$$

we deduce from $\Vert I(t)\Vert _{L_1(J,L_q(\Omega ))}\le c_0(T)$ for $t\in [0,T]$ that

$$\begin{aligned} \Vert S(t)\Vert _{L_r(\Omega )}&\le \Vert S_0\Vert _{L_r(\Omega )}+c \int _0^t\Vert e^{(t-\tau )\Delta _\mathcal {B}}\Vert _{\mathcal {L}(L_\infty (\Omega ))} \,\left\| \kappa _1\left( 1-\frac{S(\tau )}{\kappa _2}\right) S(\tau ) \right\| _{L_\infty (\Omega )} \,\textrm{d}\tau \\&\qquad + \int _0^t \Vert e^{(t-\tau )\Delta _\mathcal {B}}\Vert _{\mathcal {L}(L_q(\Omega ),L_r(\Omega ))}\,\Vert r\Vert _{L_\infty (J,L_\infty (\Omega ))}\, \Vert I(\tau )\Vert _{L_1(J,L_q(\Omega ))}\, \textrm{d}\tau \\&\le \Vert S_0\Vert _{L_r(\Omega )}+c(T) \end{aligned}$$

for $t\in [0,T]$. This proves (i).

**(ii)** Set $\frac{1}{q}:=\frac{1}{r}+\frac{1}{p}$ so that

$$\begin{aligned} \Vert B[S,I]\Vert _{L_q(\Omega )}\le \Vert b\Vert _{L_\infty (J,L_\infty (\Omega ))}\,\Vert S\Vert _{L_r(\Omega )}\, \Vert I\Vert _{L_1(J,L_p(\Omega ))}. \end{aligned}$$

Then we infer for $t\in [0,T]$ from (3.14), (3.3), and (3.5) that

$$\begin{aligned} \Vert I(t)\Vert _{L_1(J,L_p(\Omega ))}&\le \int _0^t \Vert U_A(a,0)\Vert _{\mathcal {L}(L_q(\Omega ),L_p(\Omega ))}\, \Vert B[S,I](t-a)\Vert _{L_q(\Omega )}\,\textrm{d}a\\&\qquad +\int _t^{a_m}\Vert U_A(a,a-t)\Vert _{\mathcal {L}(L_p(\Omega ))}\,\Vert I_0(a-t)\Vert _{L_p(\Omega )}\, \textrm{d}a\\&\le c(T) \int _0^t (t-\sigma )^{-\frac{n}{2r}}\, \Vert I(\sigma )\Vert _{L_1(J,L_p(\Omega ))}\,\textrm{d}\sigma +\Vert I_0\Vert _{L_1(J,L_p(\Omega ))} \end{aligned}$$

with $\frac{n}{2r}<1$ whenever $\Vert S(t)\Vert _{L_r(\Omega )}\le c_0(T)$ for $t\in [0,T]$. Hence, Gronwall’s inequality implies

$$\begin{aligned} \Vert I(t)\Vert _{L_1(J,L_p(\Omega ))}\le c(T)\big (1+\Vert I_0\Vert _{L_1(J,L_p(\Omega ))}\big ) \,,\quad t\in [0,T]\,, \end{aligned}$$

as claimed. $$\square $$

Now, since

$$\begin{aligned} \Vert I(t)\Vert _{L_1(J,L_1(\Omega ))}\le c(T),\quad t\in [0,T] \cap [0,T_m), \end{aligned}$$

by (3.18), we deduce from Lemma 3.7 (i) that

$$\begin{aligned} \Vert S(t)\Vert _{L_r(\Omega )}\le c(T),\quad t\in [0,T] \cap [0,T_m), \end{aligned}$$

for $n/2<r<n/(n-2)$ and hence

$$\begin{aligned} \Vert I(t)\Vert _{L_1(J,L_p(\Omega ))}\le c(T),\quad t\in [0,T] \cap [0,T_m), \end{aligned}$$

due to Lemma 3.7 (ii). Taking $r=q=p$ in Lemma 3.7 (i) yields now

$$\begin{aligned} \Vert S(t)\Vert _{L_p(\Omega )}\le c(T),\quad t\in [0,T] \cap [0,T_m). \end{aligned}$$

Consequently, $T_m=\infty$ according to (3.15). This completes the proof of Theorem 2.1.

## Linearized stability of steady states

We linearize (1.1) around a steady state and then derive properties of the associated linear semigroup. This allows us to introduce the notion of linear stability.

Throughout this chapter we assume (2.1) and fix an arbitrary steady state $(S_*,I_*)$ to (1.1) with regularity

4.1 $$\begin{aligned} \begin{aligned}&S_*\in W_{p,\mathcal {B}}^{2}(\Omega ), \quad S_*>0 \ \text { in }\ \Omega ,\\&I_*\in L_1(J,W_{p,\mathcal {B}}^{2}(\Omega ))\cap W_1^1(J,L_p(\Omega )),\quad I_*\ge 0 \ \text { in }\ J\times \Omega , \end{aligned} \end{aligned}$$

for some $$p>( 2\vee n)$$.

### Linearization around steady states

Linearizing (1.1) around the steady state $(S_*,I_*)$ yields the problem

4.2a $$\begin{aligned} \partial _t S(t,x)&=\Delta S(t,x)+\kappa _1S(t,x)- \dfrac{2\kappa _1 S_*(x)}{\kappa _2} S(t,x)-S(t,x)\int _0^{a_m} b(a,x)\,I_*(a,x)\,\textrm{d}a \nonumber \\&\quad -S_*(x)\int _0^{a_m} b(a,x)\,I(t,a,x)\,\textrm{d}a +\int _0^{a_m} r(a,x)\,I(t,a,x)\,\textrm{d}a \,, \end{aligned}$$

4.2b $$\begin{aligned} D I(t,a,x)&=d(a)\Delta I(t,a,x)-\big (m(a,x)+r(a,x)\big ) I(t,a,x)\,, \end{aligned}$$

4.2c $$\begin{aligned} I(t,0,x)&=S_*(x)\int _0^{a_m} b(a,x)\,I(t,a,x)\,\textrm{d}a +S(t,x)\int _0^{a_m} b(a,x)\,I_*(a,x)\,\textrm{d}a\,, \end{aligned}$$

for $(t,a,x)\in \mathbb {R}^+\times [0,a_m]\times \Omega$, and subject to the initial conditions

4.2d $$\begin{aligned} S(0,x)=S_0(x)\,,\quad I(0,a,x)&= I_0(a,x)\,,\qquad (a,x)\in (0,a_m)\times \Omega \,, \end{aligned}$$

and boundary conditions

4.2e $$\begin{aligned} \mathcal {B}S(t,x)= 0\,,\quad \mathcal {B}I(t,a,x)&= 0\,,\qquad (t,a,x)\in \mathbb {R}^+\times (0,a_m)\times \partial \Omega \,. \end{aligned}$$

Introducing

4.3a $$\begin{aligned} q_*:&=\int _0^{a_m} b(a,\cdot )\,I_*(a,\cdot )\,\textrm{d}a\in C^1({{\bar{\Omega }}})\,, \end{aligned}$$

4.3b $$\begin{aligned} P_*I&:=S_*\int _0^{a_m} b(a,\cdot )\,I(a,\cdot )\,\textrm{d}a\,,\qquad NI:=\int _0^{a_m} r(a,\cdot )\,I(a,\cdot )\,\textrm{d}a\,, \end{aligned}$$

and setting

4.3c $$\begin{aligned} A_1^*&:=\Delta _\mathcal {B} +\kappa _1- \dfrac{2\kappa _1 S_*}{\kappa _2}-q_*\,, \end{aligned}$$

4.3d $$\begin{aligned} A(a)&:=d(a)\Delta _\mathcal {B} -m(a,\cdot )-r(a,\cdot )\,,\quad a\in J\,, \end{aligned}$$

it follows

4.3e $$\begin{aligned} P_*\,,\, N\in \mathcal {L}\big (L_1(J,L_p(\Omega )),L_p(\Omega )\big ) \end{aligned}$$

and $A_1^*$ with domain $W_{p,\mathcal {B}}^2(\Omega )$ generates a positive, compact, analytic semigroup $(e^{tA_1^*})_{t\ge 0}$ on $L_p(\Omega )$ while the operator family *A*(*a*) with domain $W_{p,\mathcal {B}}^2(\Omega )$ generates a positive parabolic evolution operator $(U_{A}(a,\sigma ))_{0\le \sigma \le a\le a_m}$ on $L_p(\Omega )$. With this notation we can recast the linearization (4.2) as an equation in $L_p(\Omega )\times L_1(J,L_p(\Omega ))$ of the form

4.4a $$\begin{aligned} \partial _t\left( \begin{matrix} S \\ I \end{matrix}\right) =\left( \begin{matrix} A_1^* &{} -P_* +N \\ 0&{} -\partial _a+A(a) \end{matrix}\right) \left( \begin{matrix} S \\ I \end{matrix}\right) \,, \quad t>0\,,\qquad \left( \begin{matrix} S \\ I \end{matrix}\right) (0)=\left( \begin{matrix} S_0 \\ I_0 \end{matrix}\right) \,, \end{aligned}$$

subject to

4.4b $$\begin{aligned} I(t,0)-P_*I(t,\cdot )=q_*S(t)\,,\quad t>0\,. \end{aligned}$$

Following Walker (2021) we next show that the solutions to (4.4) are given by a strongly continuous semigroup on the phase space $L_p(\Omega )\times L_1(J,L_p(\Omega ))$.

### The semigroup associated with the linearization (4.4)

In order to investigate the properties of the semigroup generated by the linearization (4.4) we write

$$\begin{aligned} \left( \begin{matrix} A_1^* &{} -P_* +N \\ 0&{} -\partial _a+A(a) \end{matrix}\right) =\left( \begin{matrix} A_1^* &{} 0 \\ 0 &{} -\partial _a+A(a) \end{matrix}\right) +\left( \begin{matrix}0 &{} -P_* +N \\ 0&{} 0 \end{matrix}\right) \end{aligned}$$

and use a perturbation argument, first focusing on the diagonal part. In the following, we will require information on the operator family $S_*Q^\lambda$ with

4.5 $$\begin{aligned} Q^\lambda :=\int _0^{a_m} b(a)\, U_{A}^\lambda (a,0)\,\textrm{d}a,\quad \lambda \in \mathbb {C}, \end{aligned}$$

where

$$\begin{aligned} U_{A}^\lambda (a,\sigma ):=e^{-\lambda (a-\sigma )}\,U_{A}(a,\sigma ),\quad 0\le \sigma \le a\le a_m. \end{aligned}$$

It follows from (3.5) and (2.1) that

4.6 $$\begin{aligned} S_*Q^\lambda \in \mathcal {L}\big (L_p(\Omega ),W_{p,\mathcal {B}}^{1}(\Omega )\big ) \cap \mathcal {K}\big (L_p(\Omega )\big ), \end{aligned}$$

where the compact embedding of $W_{p,\mathcal {B}}^{1}(\Omega )$ into $L_p(\Omega )$ ensures the compactness of the operator $S_*Q^\lambda$ on $L_p(\Omega )$. Moreover, for $\lambda \in \mathbb {R}$, the operator $S_*Q^\lambda \in \mathcal {L}(L_p(\Omega ))$ is an irreducible operator for $p>n$ (Daners and Koch Medina (1992, Corollary 13.6)). Its spectral radius is thus characterized by the Krein-Rutman Theorem. We cite the following result in this context:

#### Lemma 4.1

(*Walker* (2013, Lemma 2.4, Lemma 2.5)) For $\lambda \in \mathbb {R}$, the spectral radius $r(S_*Q^\lambda )$ is positive and a simple eigenvalue of $\hbox {S}_*{{Q}}^\lambda \in \mathcal {K}({{L}}_{{p}}(\Omega ))$ with an eigenvector $\zeta _\lambda \in W_{p,\mathcal {B}}^1(\Omega )$ that is quasi-interior in $L_p^+(\Omega )$. It is the only eigenvalue of $S_*Q^\lambda$ with a positive eigenvector. The mapping

$$\begin{aligned} \mathbb {R}\rightarrow (0,\infty ),\quad \lambda \mapsto r(S_*Q^\lambda ) \end{aligned}$$

is continuous and strictly decreasing with

$$\begin{aligned} \lim _{\lambda \rightarrow -\infty }r(S_*Q^\lambda ) =\infty ,\qquad \lim _{\lambda \rightarrow \infty }r(S_*Q^\lambda ) =0. \end{aligned}$$

Now, in order to introduce the semigroup associated with (4.4), we recall from Walker (2021, Lemma 5.1) that there exists a mapping

$$\begin{aligned} B: [(S_0,I_0)\mapsto B_{(S_0,I_0)}]\in \mathcal {L}\big (L_p(\Omega )\times L_1(J,L_p(\Omega )), C(\mathbb {R}^+,L_p(\Omega ))\big ) \end{aligned}$$

such that $B=B_{(S_0,I_0)}$ is for given $(S_0,I_0)\in L_p(\Omega )\times L_1(J,L_p(\Omega ))$ the unique solution to the Volterra equation^2^

4.7a $$\begin{aligned} B(t)= & {} S_*\int _0^t \, b(a)\, U_{A}(a,0)\, B(t-a)\, \textrm{d}a \nonumber \\{} & {} + S_*\int _0^{a_m-t} b(a+t)\, U_{A}(a+t,a)\, I_0(a)\, \textrm{d}a +q_*e^{t A_1^*} S_0\,, \end{aligned}$$

for $t\ge 0$. If $(S_0,I_0)\in L_p^+(\Omega )\times L_1^+(J,L_p(\Omega ))$, then $B_{(S_0,I_0)}(t)\in L_p^+(\Omega )$ for $t\ge 0$. Now, given $(S_0,I_0)\in L_p(\Omega )\times L_1(J,L_p(\Omega ))$, define

4.7b $$\begin{aligned} \big [\mathbb {I}_*(t) (S_0,I_0)\big ](a)\,:=\, \left\{ \begin{aligned}&U_{A}(a,a-t)\, I_0(a-t)\,,{} & {} (a,t)\in J\times \mathbb {R}^+,\quad t\le a, \\&U_{A}(a,0)\, B_{(S_0,I_0)}(t-a)\,,{} & {} (a,t)\in J\times \mathbb {R}^+,\quad t>a\,, \end{aligned} \right. \nonumber \\ \end{aligned}$$

and

4.7c $$\begin{aligned} \mathbb {T}_*(t) (S_0,I_0)\,:=\, \big (e^{t A_1^*} S_0,\, \mathbb {I}_*(t) (S_0,I_0)\big )\,, \qquad t\ge 0. \end{aligned}$$

Then $(\mathbb {T}_*(t))_{t\ge 0}$ defines a strongly continuous semigroup on $L_p(\Omega )\times L_1(J,L_p(\Omega ))$:

#### Theorem 4.2

Suppose (2.1) and let $(S_*,I_*)$ be a steady state to (1.1) satisfying (4.1). Define $(\mathbb {T}_*(t))_{t\ge 0}$ on $L_p(\Omega )\times L_1(J,L_p(\Omega ))$ with $p>n$ according to (4.7).

**(a)** $(\mathbb {T}_*(t))_{t\ge 0}$ is a strongly continuous, eventually compact, positive semigroup on the space $L_p(\Omega )\times L_1(J,L_p(\Omega ))$.

**(b)** Let $\mathbb {A}_*$ be the infinitesimal generator of the semigroup $(\mathbb {T}_*(t))_{t\ge 0}$. Then $(\phi ,\psi )\in \textrm{dom}(\mathbb {A}_*)$ if and only if $(\phi ,\psi )\in W_{p,\mathcal {B}}^{2}(\Omega )\times C(J,L_p(\Omega ))$ and there exists $\zeta \in L_1(J,L_p(\Omega ))$ such that $\psi$ is the mild solution to

4.8a $$\begin{aligned} \partial _a\psi = A(a)\psi +\zeta (a),\quad a\in J,\qquad \psi (0)=S_*\int _0^{a_m} b(a) \psi (a)\,\textrm{d}a + q_*\phi . \end{aligned}$$

In this case,

4.8b $$\begin{aligned} \mathbb {A}_* ( \phi , \psi ) = \left( A_1^*\phi , -\zeta \right) . \end{aligned}$$

**(c)** $\mathbb {A}_*$ has compact resolvent.

**(d)** The spectral bound $s(\mathbb {A}_*)$ and the growth bound $\omega _0(\mathbb {T}_*)$ are equal.

#### Proof

**(a) Semigroup:** One may follow the lines of the proof of Webb (2008, Theorem 4) to show that $(\mathbb {T}_*(t))_{t\ge 0}$ defines a strongly continuous positive semigroup on $L_p(\Omega )\times L_1(J,L_p(\Omega ))$ (see also Walker 2013, 2021). That this semigroup is eventually compact follows as in Walker (2021, Theorem 1.2 (a)) invoking Kolmogorov’s compactness criterion and using the compact embedding of $W_{p,\mathcal {B}}^2(\Omega )$ into $L_p(\Omega )$.

**(b) Generator:** The proof of the characterization of the generator $\mathbb {A}_*$ is mostly along the lines of the proof of Walker (2021, Theorem 1.4 (a)), though a bit tricky. We provide some details here. The key is to derive a description of the resolvent $(\lambda -\mathbb {A}_*)^{-1}$.

**(i)** To this end, we fix $\lambda >0$ large enough (in particular in the resolvent set of $\mathbb {A}_*$) and write

$$\begin{aligned} (\lambda -\mathbb {A}_*)^{-1}(S_0,I_0)=(\phi ,\psi )\in L_p(\Omega )\times L_1(J,L_p(\Omega )) \end{aligned}$$

for $(S_0,I_0)\in L_p(\Omega )\times L_1(J,L_p(\Omega ))$. Then, using the Laplace transform formula

$$\begin{aligned} (\lambda -\mathbb {A}_*)^{-1}(S_0,I_0)=\int _0^\infty e^{-\lambda t}\, \mathbb {T}_*(t) (S_0,I_0)\, \textrm{d}t \end{aligned}$$

and recalling (4.7c), we readily obtain

$$\begin{aligned} \phi =\int _0^\infty e^{-\lambda t}\, e^{tA_1^*} S_0\, \textrm{d}t=(\lambda -A_1^*)^{-1}S_0 \end{aligned}$$

and using (4.7b), for $a\in J$,

4.9 $$\begin{aligned} \psi (a)= & {} \int _0^\infty e^{-\lambda t}\, \big [\mathbb {I}_*(t) (S_0,I_0)\big ](a)\, \textrm{d}t\nonumber \\= & {} \int _0^a U_{A}^\lambda (a,t)\,I_0(t)\, \textrm{d}t +U_{A}^\lambda (a,0) \int _0^\infty e^{-\lambda t} B_{(S_0,I_0)}(t)\, \textrm{d}t. \end{aligned}$$

Invoking (4.9), (4.7a), and (4.5) we derive in particular that

$$\begin{aligned} \begin{aligned} \psi (0)&=\int _0^\infty e^{-\lambda t} B_{(S_0,I_0)}(t)\, \textrm{d}t\\&=\int _0^\infty e^{-\lambda t}\, S_*\int _0^{\min \{t,a_m\}} \, b(a)\, U_{A}(a,0)\, B_{(S_0,I_0)}(t-a)\, \textrm{d}a\,\textrm{d}t\\&\quad + \int _0^{a_m} e^{-\lambda t}S_*\int _0^{a_m-t} b(a+t)\, U_{A}(a+t,a)\, I_0(a)\, \textrm{d}a\,\textrm{d}t +\int _0^\infty e^{-\lambda t}q_*e^{t A_1^*} S_0\,\textrm{d}t\\&= S_*\int _0^{a_m} b(a)\, U_{A}^\lambda (a,0)\,\textrm{d}a \,\psi (0) \\&\quad + S_*\int _0^{a_m} b(a)\int _0^a U_{A}^\lambda (a,t)\, I_0(t) \,\textrm{d}t\,\textrm{d}a +q_*(\lambda -A_1^*)^{-1}S_0\\&= S_*Q^\lambda \psi (0) + S_*\int _0^{a_m} b(a)\int _0^a U_{A}^\lambda (a,t)\, I_0(t) \,\textrm{d}t\,\textrm{d}a +q_*\phi . \end{aligned} \end{aligned}$$

Summarizing, we have shown that if $(S_0,I_0)\in L_p(\Omega )\times L_1(J,L_p(\Omega ))$ and

$$\begin{aligned} (\phi ,\psi )=(\lambda -\mathbb {A}_*)^{-1}(S_0,I_0) \end{aligned}$$

for $\lambda >0$ large enough, then

4.10a $$\begin{aligned} \phi =(\lambda -A_1^*)^{-1}S_0\in W_{p,\mathcal {B}}^2(\Omega ) \end{aligned}$$

and $\psi \in C(J,L_p(\Omega ))$ is a mild solution to

4.10b $$\begin{aligned} \partial _a\psi =(-\lambda +A(a))\psi +I_0(a),\quad a\in J, \end{aligned}$$

subject to

4.10c $$\begin{aligned} \begin{aligned} \psi (0)&= S_*\int _0^{a_m} b(a) \psi (a)\,\textrm{d}a+q_*\phi \\&=S_*Q^\lambda \psi (0) + S_*\int _0^{a_m} b(a)\int _0^a U_{A}^\lambda (a,t)\, I_0(t) \,\textrm{d}t\,\textrm{d}a +q_*\phi . \end{aligned} \end{aligned}$$

**(ii)** Consider now an arbitrary $(\phi ,\psi )\in \textrm{dom}(\mathbb {A}_*)\subset L_p(\Omega )\times L_1(J,L_p(\Omega ))$. Defining (for $\lambda >0$ large enough)

$$\begin{aligned} (S_0,I_0):=(\lambda -\mathbb {A}_*)(\phi ,\psi )\in L_p(\Omega )\times L_1(J,L_p(\Omega )), \end{aligned}$$

it readily follows from (4.10) that

$$\begin{aligned} \phi =(\lambda -A_1^*)^{-1}S_0\in W_{p,\mathcal {B}}^{2}(\Omega ) \end{aligned}$$

while $\psi \in C(J,L_p(\Omega ))$ is a mild solution to

$$\begin{aligned} \partial _a\psi =(-\lambda +A(a))\psi +I_0(a)=A(a)\psi +\zeta (a),\quad a\in J,\qquad \end{aligned}$$

with $\zeta :=I_0-\lambda \psi \in L_1(J,L_p(\Omega ))$ and subject to

4.11 $$\begin{aligned} \psi (0)= S_*\int _0^{a_m} b(a) \psi (a)\,\textrm{d}a+q_*\phi . \end{aligned}$$

This is (4.8a), while (4.8b) follows from

$$\begin{aligned} \mathbb {A}_*(\phi ,\psi )=\lambda (\phi ,\psi )-(S_0,I_0)=(A_1^*\phi , -\zeta ). \end{aligned}$$

**(iii)** Conversely, consider $(\phi ,\psi )\in W_{p,\mathcal {B}}^{2}(\Omega )\times C(J,L_p(\Omega ))$ with the property that there exists $\zeta \in L_1(J,L_p(\Omega ))$ such that $\psi$ is the mild solution to

$$\begin{aligned} \partial _a\psi = A(a)\psi +\zeta (a),\quad a\in J,\qquad \psi (0)=S_*\int _0^{a_m} b(a) \psi (a)\,\textrm{d}a + q_*\phi . \end{aligned}$$

Thus, for $\lambda >0$ large enough and

$$\begin{aligned} I_0:=\lambda \psi +\zeta \in L_1(J,L_p(\Omega )), \end{aligned}$$

we see that $\psi \in C(J,L_p(\Omega ))$ is the mild solution to

$$\begin{aligned} \partial _a\psi = (-\lambda +A(a))\psi +I_0(a),\quad a\in J,\qquad \psi (0)=S_*\int _0^{a_m} b(a) \psi (a)\,\textrm{d}a + q_*\phi , \end{aligned}$$

and thus satisfies

4.12 $$\begin{aligned} \psi (0)=S_*Q^\lambda \psi (0) + S_*\int _0^{a_m} b(a)\int _0^a U_{A}^\lambda (a,t)\, I_0(t) \,\textrm{d}t\,\textrm{d}a +q_*\phi . \end{aligned}$$

Define now

$$\begin{aligned} S_0:=(\lambda -A_1^*)\phi \in L_p(\Omega ) \end{aligned}$$

and

$$\begin{aligned} ({{\bar{\phi }}},{{\bar{\psi }}}):=(\lambda -\mathbb {A}_*)^{-1}(S_0,I_0)\in \textrm{dom}(\mathbb {A}_*). \end{aligned}$$

Then, according to (4.10),

$$\begin{aligned} {{\bar{\phi }}}=(\lambda -A_1^*)^{-1}S_0=\phi \end{aligned}$$

while ${{\bar{\psi }}}\in C(J,L_p(\Omega ))$ is the mild solution to

$$\begin{aligned} \partial _a{{\bar{\psi }}} =(-\lambda +A(a)){{\bar{\psi }}}+I_0(a),\quad a\in J, \end{aligned}$$

subject to

4.13 $$\begin{aligned} {{\bar{\psi }}}(0)&=S_*Q^\lambda {{\bar{\psi }}}(0) + S_*\int _0^{a_m} b(a)\int _0^a U_{A}^\lambda (a,t)\, I_0(t) \,\textrm{d}t\,\textrm{d}a +q_*{{\bar{\phi }}}\,. \end{aligned}$$

Since ${{\bar{\phi }}}=\phi$, it follows from (4.12) and (4.13) that

$$\begin{aligned} (1-S_*Q^\lambda ){{\bar{\psi }}}(0)=(1-S_*Q^\lambda )\psi (0) \end{aligned}$$

and hence ${{\bar{\psi }}}(0)=\psi (0)$ according to Lemma 4.1 for $\lambda >0$ large enough (so that $r(S_*Q^\lambda )<1$). Consequently, ${{\bar{\psi }}}=\psi$ and therefore

$$\begin{aligned} (\phi ,\psi )=({{\bar{\phi }}},{{\bar{\psi }}})\in \textrm{dom}(\mathbb {A}_*). \end{aligned}$$

This proves part **(b)**.

**(c) Compact Resolvent:** In order to show that $\mathbb {A}_*$ has compact resolvent, let $\lambda >0$ again be sufficiently large (i.e. $\lambda$ in the resolvent set of $\mathbb {A}_*$ and $r(S_*Q^\lambda )<1$). Let $(S_{0,j},I_{0,j})_{j\in \mathbb {N}}$ be a bounded sequence in $L_p(\Omega )\times L_1(J,L_p(\Omega ))$ and set

$$\begin{aligned} (\phi _j,\psi _j):=(\lambda -\mathbb {A}_*)^{-1}(S_{0,j},I_{0,j}). \end{aligned}$$

Then (4.10a) yields $\phi _j=(\lambda -A_1^*)^{-1}S_{0,j}$ so that $(\phi _j)_{j\in \mathbb {N}}$ is a bounded sequence in $W_{p,\mathcal {B}}^2(\Omega )$, the latter being compactly embedded in $L_p(\Omega )$. It remains to show that $(\psi _j)_{j\in \mathbb {N}}$ is relatively compact in $L_1(J,L_p(\Omega ))$ for which we first note from (4.10b)-(4.10c) that

4.14 $$\begin{aligned} \psi _j(a) =U_A^\lambda (a,0)\psi _j(0)+\int _0^aU_A^\lambda (a,\sigma ) I_{0,j}(\sigma )\,\textrm{d}\sigma ,\quad a\in J, \end{aligned}$$

and

4.15 $$\begin{aligned} (1-S_*Q^\lambda )\psi _j(0)= S_*\int _0^{a_m} b(a)\int _0^a U_{A}^\lambda (a,\sigma )\, I_{0,j}(\sigma ) \,\textrm{d}\sigma \,\textrm{d}a +q_*\phi _j. \end{aligned}$$

In particular, since by (2.1d) and (3.5)

$$\begin{aligned} \Bigg \Vert \int _0^{a_m} b(a)&\int _0^a U_{A}^\lambda (a,\sigma )\, I_{0,j}(\sigma ) \,\textrm{d}\sigma \,\textrm{d}a \Bigg \Vert _{W_{p,\mathcal {B}}^{1}(\Omega )} \\&\le c \Vert b\Vert _{L_\infty (J,C^1(\Omega ))}\, \int _0^{a_m} \int _0^a (a-\sigma )^{-1/2}\, \Vert I_{0,j}(\sigma ) \Vert _{L_p(\Omega )}\,\textrm{d}\sigma \,\textrm{d}a\\&\le c_1 \int _0^{a_m} \Vert I_{0,j}(\sigma ) \Vert _{L_p(\Omega )} \int _\sigma ^{a_m} (a-\sigma )^{-1/2} \,\textrm{d}a\,\textrm{d}\sigma \\ {}&\le c_2 \Vert I_{0,j} \Vert _{L_1(J,L_p(\Omega ))}\,, \end{aligned}$$

the sequence

$$\begin{aligned} \left( \int _0^{a_m} b(a)\int _0^a U_{A}^\lambda (a,\sigma )\, I_{0,j}(\sigma ) \,\textrm{d}\sigma \,\textrm{d}a\right) _{j\in \mathbb {N}} \end{aligned}$$

is bounded in $W_{p,\mathcal {B}}^{1}(\Omega )$, and we thus deduce from (4.15), (4.6), and $r(S_*Q^\lambda )<1$ that

4.16 $$\begin{aligned} (\psi _j(0))_{j\in \mathbb {N}}\ \text { is bounded in}\ W_{p,\mathcal {B}}^{1}(\Omega ). \end{aligned}$$

Setting

$$\begin{aligned} u_j(a):=\int _0^aU_A^\lambda (a,\sigma ) I_{0,j}(\sigma )\,\textrm{d}\sigma ,\quad a\in J,\quad j\in \mathbb {N}, \end{aligned}$$

we next show that $\{u_j;\, j\in \mathbb {N}\}$ is relatively compact in $L_1(J,L_p(\Omega ))$ adopting the arguments from Baras et al. (1977) (there the case of semigroups was considered):

**(i)** We first fix $\mu >0$ and define

$$\begin{aligned} v_j^\mu (a):=U_A^\lambda (\mu +a,a)u_j(a)=\int _0^aU_A^\lambda (\mu +a,\sigma ) I_{0,j}(\sigma )\,\textrm{d}\sigma ,\quad a\in J,\quad j\in \mathbb {N}. \end{aligned}$$

Since

$$\begin{aligned} \Vert u_j(a)\Vert _{L_p(\Omega )}\le \Vert I_{0,j}\Vert _{L_1(J,L_p(\Omega ))}, \quad a\in J,\quad j\in \mathbb {N}, \end{aligned}$$

and

$$\begin{aligned} U_A^\lambda (\mu +a,a)\in \mathcal {L}\big (L_p(\Omega ),W_{p,\mathcal {B}}^2(\Omega )\big )\subset \mathcal {K}\big (L_p(\Omega )\big ),\quad \quad a\in J, \end{aligned}$$

we see that, for every $a\in J$, the sequence $(v_j^\mu (a))_{j\in \mathbb {N}}$ is relatively compact in $L_p(\Omega )$. Next, in order to check equi-integrability we recall from Amann (1995, II. Equation (2.1.2)) that

$$\begin{aligned} U_A^\lambda \in C\big (\Delta _J^*,\mathcal {L}(L_p(\Omega ))\big ) \ \text { with }\ \Delta _J^*:=\{(a,\sigma );\, 0\le \sigma < a\le a_m\}, \end{aligned}$$

and note for $\xi >0$ that the set

$$\begin{aligned} K_\xi :=\{(a,\sigma )\in \Delta _J^*;\, \sigma +\xi \le a\} \end{aligned}$$

is compact in $\Delta _J^*$. We thus find for every $\varepsilon >0$ and $\xi >0$ some $\eta >0$ such that

$$\begin{aligned} \Vert U_A(a_1,\sigma _1)-U_A(a_2,\sigma _2)\Vert _{\mathcal {L}(L_p(\Omega ))}\le \varepsilon ,\ (a_i,\sigma _i)\in K_\xi ,\ \vert (a_1,\sigma _1)-(a_2,\sigma _2)\vert \le \eta . \end{aligned}$$

Taking $\varepsilon >0$ arbitrary and $\xi =\mu >0$, we use (3.3) to derive, for $h\in (0,\eta )$ with $0<a\le a+h\le a_m$,

$$\begin{aligned}&\Vert v_j^\mu (a+h)-v_j^\mu (a)\Vert _{L_p(\Omega )} \\ {}&\qquad \quad \le \int _a^{a+h}\Vert U_A^\lambda (\mu +a+h,\sigma )\Vert _{\mathcal {L}(L_p(\Omega ))}\,\Vert I_{0,j}(\sigma )\Vert _{L_p(\Omega )}\,\textrm{d}\sigma \\&\qquad \qquad + \int _0^{a}\Vert U_A^\lambda (\mu +a+h,\sigma )-U_A^\lambda (\mu +a,\sigma )\Vert _{\mathcal {L}(L_p(\Omega ))}\,\Vert I_{0,j}(\sigma )\Vert _{L_p(\Omega )}\,\textrm{d}\sigma \\&\qquad \quad \le \int _a^{a+h}\Vert I_{0,j}(\sigma )\Vert _{L_p(\Omega )}\,\textrm{d}\sigma +\varepsilon \int _0^{a}\Vert I_{0,j}(\sigma )\Vert _{L_p(\Omega )}\,\textrm{d}\sigma \end{aligned}$$

and therefore

$$\begin{aligned} \int _0^{a_m}\Vert {\tilde{v}}_j^\mu (a+h)-\tilde{v}_j^\mu (a)\Vert _{L_p(\Omega )}\,\textrm{d}a&\le h \Vert I_{0,j} \Vert _{L_1(J,L_p(\Omega ))}+\varepsilon a_m \Vert I_{0,j} \Vert _{L_1(J,L_p(\Omega ))}\,, \end{aligned}$$

where the tilde refers to the trivial extension. Hence,

$$\begin{aligned} \lim _{h\rightarrow 0}\, \sup _{j\in \mathbb {N}}\int _0^{a_m}\Vert \tilde{v}_j^\mu (a+h)-{\tilde{v}}_j^\mu (a)\Vert _{L_p(\Omega )}\,\textrm{d}a =0 \end{aligned}$$

so that $\{v_j^\mu ;\, j\in \mathbb {N}\}$ is equi-integrable and thus

4.17 $$\begin{aligned} \{v_j^\mu \,;\, j\in \mathbb {N}\}\ \text { is relatively compact in~} L_1(J,L_p(\Omega ))~ \text {for}~ \mu >0 \,. \end{aligned}$$

**(ii)** We next consider the limit $\mu \rightarrow 0$. Given $\varepsilon >0$, $\xi >0$, and using the notation from the previous part we have, for $a\in J$ with $a\ge \xi$ and $0<\mu <\eta$,

$$\begin{aligned}&\Vert v_j^\mu (a)-u_j(a)\Vert _{L_p(\Omega )}\\&\qquad \le \int _0^{a-\xi }\Vert U_A^\lambda (\mu +a,\sigma )-U_A^\lambda (a,\sigma )\Vert _{\mathcal {L}(L_p(\Omega ))}\,\Vert I_{0,j}(\sigma )\Vert _{L_p(\Omega )}\,\textrm{d}\sigma \\&\qquad \quad + \int _{a-\xi }^a \big (\Vert U_A^\lambda (\mu +a,\sigma )\Vert _{\mathcal {L}(L_p(\Omega ))}+\Vert U_A^\lambda (a,\sigma )\Vert _{\mathcal {L}(L_p(\Omega ))}\big )\,\Vert I_{0,j}(\sigma )\Vert _{L_p(\Omega )}\,\textrm{d}\sigma \\&\qquad \le \varepsilon \int _0^{a-\xi } \Vert I_{0,j}(\sigma )\Vert _{L_p(\Omega )}\,\textrm{d}\sigma + 2\int _{a-\xi }^a \Vert I_{0,j}(\sigma )\Vert _{L_p(\Omega )}\,\textrm{d}\sigma \,, \end{aligned}$$

while for $0\le a\le \xi$ we have

$$\begin{aligned} \Vert v_j^\mu (a)-u_j(a)\Vert _{L_p(\Omega )}&\le 2\int _0^{\xi } \Vert I_{0,j}(\sigma )\Vert _{L_p(\Omega )}\,\textrm{d}\sigma \,. \end{aligned}$$

Therefore,

$$\begin{aligned} \Vert v_j^\mu -u_j\Vert _{L_1(J,L_p(\Omega ))}&\le 4 \xi \Vert I_{0,j}\Vert _{L_1(J,L_p(\Omega ))}+\varepsilon a_m \Vert I_{0,j}\Vert _{L_1(J,L_p(\Omega ))} \end{aligned}$$

so that

$$\begin{aligned} \lim _{\mu \rightarrow 0}\, \sup _{j\in \mathbb {N}}\, \Vert v_j^\mu -u_j\Vert _{L_1(J,L_p(\Omega ))}=0\,. \end{aligned}$$

Together with (4.17) we conclude that $\{u_j;\, j\in \mathbb {N}\}$ is relatively compact in $L_1(J,L_p(\Omega ))$.

**(iii)** Finally, since

$$\begin{aligned} \Vert U_A^\lambda (a+h,0)-U_A^\lambda (a,0)\Vert _{\mathcal {L}(W_{p,\mathcal {B}}^{1}(\Omega ),L_p(\Omega ))}\le c h^{1/2},\quad 0\le a\le a+h\le a_m, \end{aligned}$$

according to Amann (1995, II. Equation (5.3.8)), it readily follows from (4.16) and the Arzelà - Ascoli Theorem that $(U_A^\lambda (\cdot ,0)\psi _j(0))_{j\in \mathbb {N}}$ is relatively compact in $C(J,L_p(\Omega ))$. Consequently, we deduce from the previous step **(ii)** and (4.14) that $(\psi _j)_{j\in \mathbb {N}}$ is relatively compact in $L_1(J,L_p(\Omega ))$. Therefore, $\mathbb {A}_*$ has indeed a compact resolvent.

**(d) Spectral Bound:** Since $\mathbb {T}_*$ is eventually compact, it follows from Engel and Nagel (2000, IV. Corollary 3.12) that $s(\mathbb {A}_*)=\omega _0(\mathbb {T}_*)$.

$$\square $$

Introducing now the perturbation

$$\begin{aligned} \mathbb {B}_*:=\left( \begin{matrix}0 &{} -P_* +N\\ 0&{}0 \end{matrix}\right) \in \mathcal {L}\big (L_p(\Omega )\times L_1(J,L_p(\Omega ))\big ) \end{aligned}$$

with $P_*$ and *N* defined in (4.3b) and observing from (4.8) that $\mathbb {A}_*+\mathbb {B}_*$ is exactly the linearized operator appearing in (4.4a) subject to (4.4b), we obtain the desired generation result for the linearization:

#### Corollary 4.3

Let the assumptions of Theorem 4.2 be satisfied. Then, the generator $\mathbb {A}_*+\mathbb {B}_*$ of the strongly continuous semigroup $(\mathbb {S}_*(t))_{t\ge 0}$ on $L_p(\Omega )\times L_1(J,L_p(\Omega ))$, associated with the linearized problem (4.4), has compact resolvent. In particular, the spectrum $\sigma (\mathbb {A}_*+\mathbb {B}_*)$ is a pure point spectrum without finite accumulation point.

#### Proof

This follows from Theorem 4.2 and Engel and Nagel (2000, III. Proposition 1.12). $\square$

#### Definition 4.4

We call the steady state $(S_*,I_*)$ *linearly stable* in the space $L_p(\Omega )\times L_1(J,L_p(\Omega ))$, if the generator $\mathbb {A}_*+\mathbb {B}_*$ of the linearized problem satisfies

$$\textrm{Re}\, \sigma (\mathbb {A}_*+\mathbb {B}_*)<0,$$

while we call $(S_*,I_*)$ *linearly unstable* if

$$ \sigma (\mathbb {A}_*+\mathbb {B}_*)\cap [\textrm{Re}\,\lambda >0]\not =\emptyset .$$

#### Remark 4.5

Corollary 4.3 implies that the linear stability of the steady state $(S_*,I_*)$ is determined from (the real parts of) those $\lambda \in \mathbb {C}$ for which there is a nontrivial $\displaystyle ({{ S,I}})\in {{ W}}_{p,\mathcal {B}}^{2}(\Omega )\times {{ C(J,L}}_p(\Omega ))$ satisfying

$$\begin{aligned} \left( \begin{matrix} A_1^* &{} -P_* +N \\ 0&{} -\partial _a+A(a) \end{matrix}\right) \left( \begin{matrix} S \\ I \end{matrix}\right) =\lambda \left( \begin{matrix} S \\ I \end{matrix}\right) \,,\qquad I(0)-P_*I=q_*S\,, \end{aligned}$$

(equality for the second component in the sense of Theorem 4.2) with notation introduced in (4.3).

#### Remark 4.6

Since the semigroup $(e^{t\mathbb {A}_*})_{t\ge 0}$ is eventually compact and due to the particular (nonlocal) form of the perturbation $\mathbb {B}_*$, one can in fact show that the perturbation semigroup $\mathbb {S}_*=(e^{t(\mathbb {A}_*+\mathbb {B}_*)})_{t\ge 0}$ is also eventually compact. Consequently,

$$\begin{aligned} \omega (\mathbb {S}_*)=s(\mathbb {A}_*+\mathbb {B}_*), \end{aligned}$$

that is, the growth bound of the semigroup $\mathbb {S}_*$ and the spectral bound of its generator $\mathbb {A}_*+\mathbb {B}_*$ coincide. A steady state $(S_*,I_*)$ is thus linearly stable in the sense of Definition 4.4 if and only if the semigroup $\mathbb {S}_*$ associated with the linearization around this steady state has an exponential decay. One can further prove that this indeed implies the asymptotic stability of the steady state. The technical details of the proof follow along the lines of Walker (2023) (see also Walker and Zehetbauer (2022)).

## Linearized stability: Proof of Theorem 2.3

We shall apply the results from the previous section. In the following, we are still imposing (2.1) with $p>(2\vee n)$ and assume for simplicity (2.4). Recall that $\kappa _1, \kappa _2>0$. We consider only steady states $(S_*,I_*)$ to (1.1) with regularity as in (4.1).

For the investigation of stability of steady states recall that the spectrum of the Laplacian is (counted according to multiplicity)

$$\begin{aligned} \sigma (-\Delta _\mathcal {B})= \{\mu _0,\mu _1,\mu _2,\ldots \} \end{aligned}$$

with $0\le \mu _i\le \mu _{i+1}$ for $i\ge 0$. In fact, $\mu _0>0$ in the Dirichlet case $\delta =0$ and $\mu _0=0$ in the Neumann case $\delta =1$.

### The trivial steady state

The linear stability of the trivial steady state $(S_*,I_*)=(0,0)$ depends on the sign of $\kappa _1-\mu _0$ (which is always positive for the Neumann case $\delta =1$ but may be negative in the Dirichlet case $\delta =0$):

#### Proposition 5.1

The trivial steady state $(S_*,I_*)=(0,0)$ is linearly unstable if $\kappa _1>\mu _0$ and linearly stable if $\kappa _1<\mu _0$.

#### Proof

Using the notation introduced in (4.3) for $(S_*,I_*)=(0,0)$, we have

$$\begin{aligned} q_*=0,\qquad P_*=0,\qquad A_1^*=\Delta _\mathcal {B}+\kappa _1, \end{aligned}$$

so that Remark 4.5 leads to investigating the eigenvalue problem

$$\begin{aligned} \left( \begin{matrix} \Delta _\mathcal {B}+\kappa _1 &{} 0 \\ 0&{} -\partial _a+A(a)\end{matrix}\right) \left( \begin{matrix} S \\ I \end{matrix}\right) =\lambda \left( \begin{matrix} S \\ I \end{matrix}\right) \,,\qquad I(0)=0\,, \end{aligned}$$

for a nontrivial $(S,I)\in W_{p,\mathcal {B}}^{2}(\Omega )\times C(J,L_p(\Omega ))$; that is,

$$\begin{aligned} -\Delta _\mathcal {B}S&=(\kappa _1-\lambda ) S\,, \\ \partial _aI(a)&=\big (-\lambda +A(a)\big )I(a) \,,\quad a\in J\,,\qquad I(0)=0\,, \end{aligned}$$

with mild solution *I*. Hence $I=0$ so that $S\not =0$ and thus $\kappa _1-\lambda \in \sigma (-\Delta _\mathcal {B})$. Consequently, $s(\mathbb {A}_*)=\kappa _1-\mu _0$ is an eigenvalue. $\square$

### The disease-free steady state

The existence of a disease-free steady state $(S_*,I_*)=( \tilde{S}_*,0)$ reduces to finding a positive non-trivial solution $\tilde{S}_*\in W_{p,\mathcal {B}}^2(\Omega )$ to the semilinear equation

5.1 $$\begin{aligned} -\Delta _\mathcal {B} {\tilde{S}}_*+\frac{\kappa _1}{\kappa _2} \tilde{S}_*^2=\kappa _1 {\tilde{S}}_*. \end{aligned}$$

Clearly, in the Neumann case $\delta =1$, the positive (constant) solution to (5.1) is ${\tilde{S}}_*=\kappa _2$.

To continue let us recall the following result from Amann (2005, Theorem 12, Theorem 16):

#### Lemma 5.2

Let $q\in L_\infty (\Omega )$. Then the eigenvalue problem

$$\begin{aligned} -\Delta _\mathcal {B} u+q u=\lambda u \end{aligned}$$

has a smallest eigenvalue $\lambda =\lambda _0(q)\in \mathbb {R}$ (in the sense that $\textrm{Re}\, \lambda >\lambda _0(q)$ for every other eigenvalue $\lambda$). This principal eigenvalue is simple and the only eigenvalue with a positive eigenfunction $u_0$, i.e. $u_0\in W_{p,\mathcal {B}}^2(\Omega )$ for every $p\in (1,\infty )$ and $u_0>0$ in $\Omega$. Moreover, if $q_1,q_2\in L_\infty (\Omega )$ with $q_1\le q_2$ and $q_1\not \equiv q_2$, then $\lambda _0(q_1)<\lambda _0( q_2)$. In fact, $\lambda _0(0)=\mu _0$.

Now, if ${\tilde{S}}_*\in W_{p,\mathcal {B}}^2(\Omega )$ is a positive non-trivial solution to (5.1), then necessarily

5.2 $$\begin{aligned} \kappa _1=\lambda _0\left( \frac{\kappa _1 {\tilde{S}}_*}{\kappa _2} \right) >\lambda _0(0)=\mu _0. \end{aligned}$$

Conversely, it follows from Blat and Brown (1986) that if $\kappa _1>\lambda _0(0)=\mu _0$, then (5.1) admits a positive solution ${\tilde{S}}_*\in W_{p,\mathcal {B}}^2(\Omega )$ (see also Walker (2011)). This solution is unique. Indeed, if there was another positive solution ${\hat{S}}_*\in W_{p,\mathcal {B}}^2(\Omega )$ to (5.1), then $z:={\tilde{S}}_*-{\hat{S}}_*$ solves the eigenvalue equation

$$\begin{aligned} -\Delta _\mathcal {B} z+\frac{\kappa _1}{\kappa _2}({\tilde{S}}_*+{\hat{S}}_*) z=\kappa _1 z \end{aligned}$$

so that

$$\begin{aligned} \kappa _1\ge \lambda _0\left( \frac{\kappa _1}{\kappa _2}({\tilde{S}}_*+{\hat{S}}_*)\right) . \end{aligned}$$

According to (5.2) and the monotonicity of $\lambda _0(q)$ with respect to *q* this yields the contradiction

$$\begin{aligned} \kappa _1=\lambda _0\left( \frac{\kappa _1 {\tilde{S}}_*}{\kappa _2} \right) <\lambda _0\left( \frac{\kappa _1}{\kappa _2}({\tilde{S}}_*+{\hat{S}}_*)\right) \le \kappa _1. \end{aligned}$$

The linear stability of the disease-free steady state $(S_*,I_*)=({\tilde{S}}_*,0)$ is then determined from the value of

5.3a $$\begin{aligned} {\textsf{R}}_0:=r({\tilde{S}}_*Q^0)>0, \end{aligned}$$

where the family of compact operators

5.3b $$\begin{aligned} Q^\lambda =\int _0^{a_m} b(a)\, e^{-\lambda a}\, U_{A}(a,0)\,\textrm{d}a\in \mathcal {K}(L_p(\Omega )),\quad \lambda \in \mathbb {C}, \end{aligned}$$

was introduced in (4.5) and properties of the spectral radius $r({\tilde{S}}_*Q^\lambda )$ are stated in Lemma 4.1 for $\lambda \in \mathbb {R}$.

#### Proposition 5.3

There is a disease-free steady state $(S_*,I_*)=({\tilde{S}}_*,0)$ with a smooth function ${\tilde{S}}_*>0$ if and only if $\kappa _1>\mu _0$. In this case, ${\tilde{S}}_*$ is unique, (5.2) holds, and ${\textsf{R}}_0>0$ in (5.3a) is well-defined. For Neumann boundary conditions (i.e. $\delta =1$), we have ${\tilde{S}}_*=\kappa _2$.

Moreover, $(S_*,I_*)=({\tilde{S}}_*,0)$ is linearly stable in ${{L}}_{{p}}(\Omega )\times {{L}}_1({{J,L}}_{{p}}(\Omega ))$ if ${\textsf{R}}_0<1$ and linearly unstable if  ${\textsf{R}}_0>1$.

#### Proof

We have already shown that there is a (unique) disease-free steady state $(S_*,I_*)=({\tilde{S}}_*,0)$ with ${\tilde{S}}_*>0$ (satisfying (5.2)) if and only if $\kappa _1>\mu _0$. Thus, let $\kappa _1>\mu _0$. According to Remark 4.5 we have to check the real parts of solutions $\lambda$ to the eigenvalue problem

$$\begin{aligned} \left( \begin{matrix} A_1^* &{} -P_* \\ 0&{} -\partial _a+A(a) \end{matrix}\right) \left( \begin{matrix} S \\ I \end{matrix}\right) =\lambda \left( \begin{matrix} S \\ I \end{matrix}\right) \,,\qquad I(0)=P_*I\,, \end{aligned}$$

with a nontrivial $(S,I)\in W_{p,\mathcal {B}}^{2}(\Omega )\times C(J,L_p(\Omega ))$, where, due to (4.3),

$$\begin{aligned}{} & {} q_*=0,\quad P_*I={\tilde{S}}_*\int _0^{a_m}b(a,\cdot )I(a,\cdot )\,\textrm{d}a, \\ {}{} & {} A_1^*=\Delta _\mathcal {B}+\kappa _1-\frac{2\kappa _1 \tilde{S}_*}{\kappa _2},\quad \\{} & {} A(a)=d(a)\Delta _\mathcal {B}-m(a,\cdot ). \end{aligned}$$

That is, we have to investigate

5.4 $$\begin{aligned}&-\Delta _\mathcal {B}S+\frac{2\kappa _1 {\tilde{S}}_*}{\kappa _2} S=(\kappa _1-\lambda )S-{\tilde{S}}_*Q^\lambda I(0)\,, \end{aligned}$$

5.5 $$\begin{aligned}&\partial _aI=(-\lambda +A(a))I\,,\quad a\in J\,,\qquad I(0)={\tilde{S}}_*\int _0^{a_m}b(a,\cdot )I(a,\cdot )\,\textrm{d}a \,, \end{aligned}$$

where (5.5) entails

5.6 $$\begin{aligned} I(a)=e^{-\lambda a}U_A(a,0)I(0)\,,\quad a\in J\,,\qquad I(0)=\tilde{S}_*Q^\lambda I(0)\,. \end{aligned}$$

Assume first that ${\textsf{R}}_0<1$. Then either $I(0)=0$ so that (5.4), the monotonicity of the principal eigenvalue, and (5.2) imply

$$\begin{aligned} \textrm{Re}\,(\kappa _1-\lambda )\ge \lambda _0\left( \frac{2\kappa _1 {\tilde{S}}_*}{\kappa _2}\right) > \lambda _0\left( \frac{\kappa _1 \tilde{S}_*}{\kappa _2}\right) =\kappa _1, \end{aligned}$$

hence $\textrm{Re}\,\lambda <0$. Or $I(0)\not =0$ so that (5.6) together with Walker (2021, Theorem 2.3 (b)) imply that $\lambda \in \sigma (\mathbb {A})$, where $\mathbb {A}$ is the generator of a strongly continuous, positive, eventually compact semigroup on $L_1(J,L_p(\Omega ))$. Its spectral bound $s:=s(\mathbb {A})$ is the unique real number with $r({\tilde{S}}_*Q^s)=1$ according to Walker (2021, Proposition 5.2). Since ${\textsf{R}}_0=r(\tilde{S}_*Q^0)<1$, it follows from Lemma 4.1 that $s=s(\mathbb {A})<0$ and hence again $\textrm{Re}\,\lambda <0$. Consequently, if ${\textsf{R}}_0<1$, then $(S_*,I_*)=({\tilde{S}}_*,0)$ is linearly stable.

Conversely, if ${\textsf{R}}_0=r({\tilde{S}}_*Q^0) >1$, then there is $\lambda >0$ such that $r({\tilde{S}}_*Q^\lambda )=1$ by Lemma 4.1 and there is a nontrivial $I(0)\in W_{p,\mathcal {B}}^1(\Omega )$ with $(1-{\tilde{S}}_*Q^\lambda )I(0)=0$. Then

$$\begin{aligned} I(a):= e^{-\lambda a}U_A(a,0)I(0),\quad a\in J, \end{aligned}$$

satisfies (5.5). Moreover, owing to (5.2), we have

$$\begin{aligned} \kappa _1-\lambda<\kappa _1 = \lambda _0\left( \frac{\kappa _1 \tilde{S}_*}{\kappa _2}\right) <\lambda _0\left( \frac{2\kappa _1 \tilde{S}_*}{\kappa _2}\right) , \end{aligned}$$

and thus $\kappa _1-\lambda$ belongs to the resolvent set of $-\Delta _\mathcal {B}+2\kappa _1 {\tilde{S}}_*/\kappa _2$. Hence,

$$\begin{aligned} S:=\left( -\Delta _\mathcal {B}+\frac{2\kappa _1 \tilde{S}_*}{\kappa _2}-\kappa _1+\lambda \right) ^{-1} I(0)\in W_{p,\mathcal {B}}^2(\Omega ) \end{aligned}$$

is a nontrivial solution to (5.4). That is, $\lambda >0$ is an eigenvalue and the disease-free steady state $(S_*,I_*)=(\tilde{S}_*,0)$ is thus linearly unstable. $\square$

### Non-existence of endemic steady states for ${\textsf{R}}_0\le 1$

An endemic steady state $(S_*,I_*)$ is a steady state to (1.1) with $S_*, I_*\ge 0$ and $I_*\not \equiv 0$. Note that, setting $I_0:=I_*(0)$, this is equivalent to finding a positive element $(S_*,I_0)\in W_{p,\mathcal {B}}^2(\Omega )\times W_{p,\mathcal {B}}^1(\Omega )$ with $I_0\not =0$ satisfying

5.7a $$\begin{aligned} -\Delta _\mathcal {B}S_*+ Q^0 I_0S_*+\frac{\kappa _1}{\kappa _2}S_*^2&=\kappa _1 S_*\,, \end{aligned}$$

5.7b $$\begin{aligned} I_0&=S_*Q^0 I_0\,. \end{aligned}$$

As shown next, ${\textsf{R}}_0> 1$ is a necessary condition for the existence of an endemic state.

#### Lemma 5.4

Let $\kappa _1>\mu _0$ and let ${\tilde{S}}_*$ be as in Proposition 5.3. If ${\textsf{R}}_0=r({\tilde{S}}_*Q^0)\le 1$, then there is no positive solution $(S_*,I_0)\in W_{p,\mathcal {B}}^2(\Omega )\times W_{p,\mathcal {B}}^1(\Omega )$ to  (5.7) with $I_0\not =0$.

#### Proof

Let ${\textsf{R}}_0=r({\tilde{S}}_*Q^0)\le 1$ and assume for contradiction that there was a positive solution $(S_*,I_0)\in W_{p,\mathcal {B}}^2(\Omega )\times W_{p,\mathcal {B}}^1(\Omega )$ to  (5.7) with $I_0\not =0$. It follows from (5.1) and (5.7a) that $z:=S_*-{\tilde{S}}_*$ solves

$$\begin{aligned} -\Delta _\mathcal {B}z+ Q^0 I_0z+\frac{\kappa _1}{\kappa _2}(S_*+\tilde{S}_*)z=\kappa _1 z- I_0. \end{aligned}$$

Moreover, we infer from (5.7a) and Lemma 5.2 that

$$\begin{aligned} 0<\kappa _1=\lambda _0\left( Q^0 I_0+\frac{\kappa _1}{\kappa _2} S_*\right) <\lambda _0\left( Q^0 I_0+\frac{\kappa _1}{\kappa _2} (S_*+{\tilde{S}}_*)\right) . \end{aligned}$$

Hence, $\kappa _1$ belongs to the resolvent set of the operator

$$\begin{aligned} -\Delta _\mathcal {B}+ Q^0 I_0+\frac{\kappa _1}{\kappa _2}(S_*+\tilde{S}_*) \end{aligned}$$

and consequently, since $I_0>0$,

$$\begin{aligned} z=-\left( \kappa _1-\Delta _\mathcal {B}z+ Q^0 I_0z+\frac{\kappa _1}{\kappa _2}(S_*+{\tilde{S}}_*)\right) ^{-1} I_0 \le 0\quad \text {in }\ \Omega , \end{aligned}$$

where we used the maximum principle from Amann (2005, Theorem 13). That is, $S_*\le {\tilde{S}}_*$ in $\Omega$ and thus $S_*Q^0\le {\tilde{S}}_*Q^0$. The Krein-Rutman Theorem now yields for the spectral radii

$$\begin{aligned} r(S_*Q^0)<r( {\tilde{S}}_*Q^0)\le 1, \end{aligned}$$

and therefore that the eigenvector equation $p=S_*Q^0p$ has no positive nontrivial solution in contradiction to $I_0>0$ solving (5.7b). $\square$

#### Remarks 5.5

**(a)** As noted in the proof of Lemma 5.4, necessary conditions for the existence of an endemic steady state $(S_*,I_*)$ with $S_*, I_*\ge 0$ and $I_*\not \equiv 0$ are (see (5.7))

$$\begin{aligned} \kappa _1=\lambda _0\left( Q^0 I_*(0)+\frac{\kappa _1}{\kappa _2} S_*\right) , \qquad 1=r (S_*Q^0) <r( \tilde{S}_*Q^0)={\textsf{R}}_0,\qquad S_*\le {\tilde{S}}_*, \end{aligned}$$

where $({\tilde{S}}_*,0)$ is the disease-free steady state.

**(b)** Whether the condition ${\textsf{R}}_0>1$ is sufficient for the existence of an endemic steady state is, however, left open. In fact, one can show (see Blat and Brown 1986) that for every $I_0\in L_p^+(\Omega )$ with $\lambda _0(Q^0I_0)<\kappa _1$ there is a unique solution $S_*=S_*(I_0)\in W_{p,\mathcal {B}}^2(\Omega )$ to (5.7a) with $S_*(I_0)>0$ depending compactly and smoothly on $I_0$ (by the implicit function theorem), where $\tilde{S}_*=S_*(0)$. This then reduces problem (5.7) to finding a nontrivial positive fixed point of the smooth, compact operator *F* defined by $F(I_0):=S_*(I_0) Q^0 I_0$. Noticing that $DF(0)=\tilde{S}_* Q^0$ has a positive eigenvector associated with the eigenvalue ${\textsf{R}}_0>1$ by the Krein-Rutman Theorem, it would remain to find $\rho >0$ such that $\lambda _0(Q^0I_0)<\kappa _1$ for $\Vert I_0\Vert _{L_p}\le \rho$ and $r(S_*(I_0)Q^0)<1$ when $\Vert I_0\Vert _{L_p}= \rho$. This then would allow one to apply the fixed point theorem of Amann (1976, Theorem 13.2) to derive the existence of a (unique) positive nontrivial solution $I_0=F(I_0)$ and thus an endemic steady state $(S_*(I_0),I_*)$ with $I_*(a):=U_A(a,0)I_0$. However, it is open whether this is indeed possible.

Nevertheless, when considering spatially homogeneous rates and Neumann boundary conditions, there exists a linearly stable endemic state if ${\textsf{R}}_0>1$ as stated in Theorem 2.4.

## Linearized stability in a particular model: Proof of Theorem 2.4

For Neumann boundary conditions $\delta =1$ and spatially homogeneous rates $m=m(a)$ and $b=b(a)$ we can improve the results from the previous section. Thus assume (2.4), (2.5), and $p>(2\vee n)$. Recall that the principal eigenvalue of the Laplacian $-\Delta _N$ subject to Neumann boundary conditions is $\mu _0=0$. We write $W_{p,N}^2(\Omega )$ in the following for the domain of $-\Delta _N$. Since

$$\begin{aligned} A(a)=d(a)\Delta _N -m(a)\,,\quad a\in J\,, \end{aligned}$$

and *m* is spatially homogeneous, the corresponding evolution operator is given by

6.1 $$\begin{aligned} U_{A}(a,\sigma )=\exp \left( -\int _\sigma ^a m(\tau )\,\textrm{d}\tau \right) \,\exp \left( \int _\sigma ^a d(\tau )\,\textrm{d}\tau \, \Delta _N\right) \,,\quad 0\le \sigma \le a\le a_m\,. \end{aligned}$$

In the previous section we showed that the trivial steady state $(S_*,I_*)=(0,0)$ is linearly unstable, and we have discussed the local linear stability of the disease-free steady state $(\tilde{S}_*,0)=(\kappa _2,0)$. We next investigate the latter’s *global* stability.

### Global stability of the disease-free steady state $(S_*,I_*)=(\kappa _2,0)$

We consider $\hbox {S}_{*}={\tilde{S}}_*=\kappa _{2}$. Using (6.1), the operators $Q^\lambda$ from (5.3b) become

$$\begin{aligned} Q^\lambda = \int _0^{a_m} b(a)\, e^{-\lambda a}\,\Pi (a)\, \exp \left( \int _0^a d(\tau )\,\textrm{d}\tau \, \Delta _N\right) \,\textrm{d}a,\quad \lambda \in \mathbb {C}, \end{aligned}$$

where

$$\begin{aligned} \Pi (a):=\exp \left( -\int _0^a m(\sigma )\,\textrm{d}\sigma \right) ,\quad a\in J. \end{aligned}$$

Noticing

$$\begin{aligned} {\tilde{S}}_*Q^0\textbf{1}&=\kappa _2\int _0^{a_m} b(a)\,\Pi (a) \,\textrm{d}a \, \textbf{1}\,, \end{aligned}$$

it readily follows from Lemma 4.1 for the spectral radius (see (2.6)) that

6.2 $$\begin{aligned} {\textsf{R}}_0=r({\tilde{S}}_*Q^0)=\kappa _2\int _0^{a_m} b(a) \Pi (a)\, \textrm{d}a. \end{aligned}$$

We have seen in Proposition 5.3 that $(S_*,I_*)=(\kappa _2,0)$ is linearly stable when ${\textsf{R}}_0 <1$ and linearly unstable when ${\textsf{R}}_0 >1$. In the former case, we can prove now its global stability. That is, if ${\textsf{R}}_0 <1$, then any solution to (1.1) subject to positive initial values $(S_0,I_0)$ converges to $(S_*,I_*)=(\kappa _2,0)$.

#### Proposition 6.1

Assume (2.4) and (2.5). Let ${\textsf{R}}_0 <1$. Consider any non-trivial $(S_0,I_0)\in L_p^+(\Omega )\times L_1^+(J, L_p\Omega ))$ and let (*S*, *I*) be the corresponding positive global solution to (1.1) provided by Theorem 2.1. Then

$$\begin{aligned} \lim _{t\rightarrow \infty } (S(t),I(t))=(\kappa _2,0)\ \text { in }\ L_p(\Omega )\times L_1(J, C({{\bar{\Omega }}})). \end{aligned}$$

#### Proof

Since solutions become immediately smooth according to Theorem 2.1, we may restrict without loss of generality to initial values

$$\begin{aligned} S_0\in W_{p,N}^2(\Omega ),\quad I_0\in L_1(J, W_{p,N}^2(\Omega )),\qquad S_0, I_0\ge 0,\qquad S_0\not = 0. \end{aligned}$$

**(i)** We first derive an upper bound on *S*. From (1.1a) we have

$$\begin{aligned} \partial _t S(t,x)\le \Delta _N S(t,x)+\kappa _1\left( 1-\dfrac{1}{\kappa _2}S(t,x)\right) S(t,x) \end{aligned}$$

so that

6.3 $$\begin{aligned} S(t,x)\le z(t),\quad (t,x)\in \mathbb {R}^+\times \Omega , \end{aligned}$$

by the comparison principle, where

$$\begin{aligned} z(t):=\frac{\Vert S_0\Vert _\infty }{e^{-\kappa _1 t}\left( 1-\frac{\kappa _1}{\kappa _2}\Vert S_0\Vert _\infty \right) +\frac{1}{\kappa _2}\Vert S_0\Vert _\infty },\quad t\ge 0, \end{aligned}$$

is the solution to

$$\begin{aligned} z'(t)=\kappa _1\big (1-\dfrac{1}{\kappa _2}z(t)\big ) z(t),\quad t\ge 0,\qquad z(0)=\Vert S_0\Vert _\infty . \end{aligned}$$

Due to ${\textsf{R}}_0 <1$ and (6.2) we may choose $\varepsilon _0>0$ such that

6.4 $$\begin{aligned} (\kappa _2+\varepsilon _0)\int _0^{a_m} b(a)\, \Pi (a)\,\textrm{d}a <1. \end{aligned}$$

Since $\lim _{t\rightarrow \infty }z(t)=\kappa _2$, we find for fixed $\varepsilon \in (0,\varepsilon _0)$ some $t_0>0$ with

6.5 $$\begin{aligned} S(t,x)\le \kappa _2+\varepsilon \,,\quad t\ge t_0\,,\quad x\in \Omega \,. \end{aligned}$$

**(ii)** Next, we derive an upper bound for *I*. Using (6.5) and (1.1b)-(1.1c) we obtain

$$\begin{aligned} D I(t+t_0,a)&=A(a)I(t+t_0,a) \end{aligned}$$

subject to

$$\begin{aligned} I(t+t_0,0)&\le (\kappa _2+\varepsilon ) \int _0^{a_m} b(a)\,I(t+t_0,a)\,\textrm{d}a\,, \quad t\ge 0\,. \end{aligned}$$

Let *G* solve (see Walker (2021))

6.6a $$\begin{aligned} D G(t,a)&=A(a) G(t,a)\,, \quad t\ge 0\,,\quad \quad a\in J\,, \end{aligned}$$

6.6b $$\begin{aligned} G(t,0)&= (\kappa _2+\varepsilon ) \int _0^{a_m} b(a)\,G(t,a)\,\textrm{d}a\,, \qquad G(0,a)=I(t_0,a)\,. \end{aligned}$$

We claim that

6.7 $$\begin{aligned} I(t+t_0,a)\le G(t,a)\,,\quad t\ge 0\,,\quad a\in J\,. \end{aligned}$$

Indeed, setting $w(t,a):=G(t,a)-I(t+t_0,a)$, we have, for $t\ge 0$ and $a\in J$,

$$\begin{aligned} D w(t,a)&=A(a) w(t,a) \,, \\ w(t,0)&\ge (\kappa _2+\varepsilon ) \int _0^{a_m} b(a)\,w(t,a)\,\textrm{d}a\,, \qquad w(0,a)=0\,, \end{aligned}$$

and therefore

6.8 $$\begin{aligned} w(t,a):=\left\{ \begin{array}{ll} 0\,,&{} a>t\,, \ a\in J\,,\\ U_A(a,0){\hat{B}}(t-a) \,,&{}a\le t\,,\ a\in J\,. \end{array} \right. \end{aligned}$$

with

$$\begin{aligned} {\hat{B}}(t)=w(t,0)\ge (\kappa _2+\varepsilon ) \int _0^{t} b(a)\,U_A(a,0)\,{\hat{B}}(t-a)\,\textrm{d}a,\quad t\ge 0. \end{aligned}$$

Introducing for $t\in [0,T]$ and $B\in C([0,T],L_p(\Omega ))$

$$\begin{aligned} (\mathcal {K}B)(t):= (\kappa _2+\varepsilon ) \int _0^{t} b(a)\,U_A(a,0)\,B(t-a)\,\textrm{d}a \end{aligned}$$

it follows that $\mathcal {K}\in \mathcal {L}\big (C([0,T],L_p(\Omega ))\big )$ is a positive compact (Volterra) operator with spectral radius zero (see the proof of Walker (2021, Lemma 5.1)). Therefore,

$$\begin{aligned} (1-\mathcal {K}) ^{-1}=\sum _{k\ge 0}\mathcal {K}^k\ge 0 \end{aligned}$$

and consequently

$$\begin{aligned} {\hat{B}}=(1-\mathcal {K}) ^{-1} h\ge 0 \end{aligned}$$

for

$$\begin{aligned} h(t):={\hat{B}}(t)- (\kappa _2+\varepsilon ) \int _0^{t} b(a)\,{\hat{B}}(t-a)\,\textrm{d}a\ge 0,\quad t\ge 0. \end{aligned}$$

Hence $w\ge 0$ according to (6.8) so that (6.7) is true.

**(iii)** Next, we claim that

6.9 $$\begin{aligned} I(t)\rightarrow 0 \ \text { in }\ L_1(J,C({{\bar{\Omega }}}))\ \text { as }\ t\rightarrow \infty , \end{aligned}$$

which, according to (6.7), is ensured by showing that

6.10 $$\begin{aligned} \lim _{t\rightarrow \infty } G(t)=0\ \text { in }\ L_1(J, C({{\bar{\Omega }}}))\,. \end{aligned}$$

As for (6.10) we fix $\alpha \in (n/2p,1)$ and note that the linear problem (6.6) with birth rate $(\kappa _2+\varepsilon )b(a)$ fits exactly into the setting of problems investigated in Walker (2021). In fact, since $G(0)=I(t_0)\in L_1(J,W_{p,N}^{2\alpha }(\Omega ))$, it follows from Walker (2021, Corollary 1.3) that $G(t)=e^{{t{\hat{\mathbb {A}}}}_{\varepsilon }}G(0)$, $t\ge 0$, where $(e^{{t{\hat{\mathbb {A}}}}_{\varepsilon }})_{t\ge 0}$ is an eventually compact, positive semigroup on $L_1(J,W_{p,N}^{2\alpha }(\Omega ))$. Therefore, Engel and Nagel (2000,V. Corollary 3.2) ensures that the spectrum of the generator ${\hat{\mathbb {A}}}_{{\varepsilon }}$ consists of eigenvalues only, while Engel and Nagel (2000, IV. Corollary 3.12) yields that the spectral bound of ${\hat{\mathbb {A}}}_{{\varepsilon }}$ coincides with the type of the semigroup. In fact, the spectral bound $s_0:=s({\hat{\mathbb {A}}}_{{\varepsilon }})$ is the unique real number with $r({\hat{Q}}_\varepsilon ^{s_0})=1$ according to Walker (2021, Proposition 5.2), where

$$\begin{aligned} {\hat{Q}}_\varepsilon ^\lambda :=(\kappa _2+\varepsilon ) \int _0^{a_m} b(a)\, U_A^\lambda (a,0)\,\textrm{d}a,\quad \lambda \in \mathbb {C}. \end{aligned}$$

Since as in (6.2)

$$\begin{aligned} r({\hat{Q}}_\varepsilon ^{s_0})=(\kappa _2+\varepsilon )\int _0^{a_m} b(a)\, \Pi (a) e^{-s_0a}\,\textrm{d}a , \end{aligned}$$

it follows from (6.4) that $s_0=s({\hat{\mathbb {A}}}_{{\varepsilon }})<0$, and since the spectral bound and the type of the semigroup coincide, we conclude that

$$\begin{aligned} \Vert G(t)\Vert _{L_1(J,W_{p,N}^{2\alpha }(\Omega ))}\le N\, e^{-s_0 t}\,\Vert G(0)\Vert _{L_1(J,W_{p,N}^{2\alpha }(\Omega ))}\longrightarrow 0 \end{aligned}$$

as $t\rightarrow \infty$. Consequently, since $W_{p,N}^{2\alpha }(\Omega )\hookrightarrow C({{\bar{\Omega }}})$, we deduce (6.10) and therefore, owing to (6.7), also (6.9).

**(iv)** Finally, we prove that

$$\begin{aligned} \lim _{t\rightarrow \infty } S(t)=\kappa _2 \ \text { in }\ L_p(\Omega ). \end{aligned}$$

Given $\varepsilon \in (0,\kappa _1)$ we infer from (6.9) that there is $t_1\ge t_0$ with

$$\begin{aligned} \left\| \int _0^{a_m}b(a)I(t,a)\,\textrm{d}a\right\| _\infty \le \varepsilon ,\quad t\ge t_1, \end{aligned}$$

and hence, due to (1.1a),

$$\begin{aligned} \partial _t S(t,x)&\ge \Delta _N S(t,x)+\kappa _1\left( 1-\dfrac{1}{\kappa _2}S(t,x)\right) S(t,x) -\varepsilon S(t,x)\,,\quad t\ge t_1\,,\quad x\in \Omega \,. \end{aligned}$$

Since the strong maximum principle yields for every $x_0\in \Omega$ some $\varrho >0$ such that

$$\begin{aligned} \varrho _0:=\min _{{{\bar{\mathbb {B}}}}(x_0,\varrho )} S(t_1,\cdot )>0, \end{aligned}$$

we obtain $S(t,x)\ge \xi (t)$ for $t\ge t_1$ and $x\in {{\bar{\mathbb {B}}}}(x_0,\varrho )$, where $\xi$ solves

$$\begin{aligned} \xi '(t)=\kappa _1\left( 1-\frac{\xi (t)}{\kappa _2}\right) \xi (t)-\varepsilon \xi (t),\quad t\ge t_1,\qquad \xi (t_1)=\xi _0. \end{aligned}$$

Therefore,

$$\begin{aligned} \liminf _{t\rightarrow \infty } S(t,x)\ge \lim _{t\rightarrow \infty }\xi (t)=\frac{\kappa _2(\kappa _1-\varepsilon )}{\kappa _1},\quad x\in \Omega . \end{aligned}$$

Letting $\varepsilon \rightarrow 0$ and invoking (6.5), we derive

$$\begin{aligned} \lim _{t\rightarrow \infty } S(t,x)=\kappa _2,\quad x\in \Omega . \end{aligned}$$

Finally, using again the $L_\infty$-bound from (6.5) and Lebesgue’s theorem we conclude that $S(t)\rightarrow \kappa _2$ in $L_p(\Omega )$ as $t\rightarrow \infty$. Together with (6.9), this proves Proposition 6.1. $\square$

### The endemic steady state $({{\bar{S}}}_*,{{\bar{I}}}_*)$

In case that the basic reproduction number satisfies

$$\begin{aligned} {\textsf{R}}_0=\kappa _2\int _0^{a_m} b(a) \Pi (a)\, \textrm{d}a >1\,, \end{aligned}$$

there is an endemic steady state $({{\bar{S}}}_*,{{\bar{I}}}_*)$ given by

$$\begin{aligned} {{\bar{S}}}_*:=\frac{\kappa _2}{{\textsf{R}}_0 },\qquad {{\bar{I}}}_*(a):= \frac{1}{{\textsf{R}}_0 } \kappa _1\kappa _2\left( 1-\frac{1}{{\textsf{R}}_0 }\right) \Pi (a),\quad a\in J. \end{aligned}$$

It is convenient to set ${\textsf{r}}_0:=1/{\textsf{R}}_0 \in (0,1)$. Then, the endemic steady state can be written as

$$\begin{aligned} {{\bar{S}}}_*={\textsf{r}}_0 \kappa _2,\qquad {{\bar{I}}}_*(a)=\Pi (a)i_*,\quad a\in J,\qquad i_*:={\textsf{r}}_0 \kappa _1\kappa _2\left( 1-{\textsf{r}}_0 \right) . \end{aligned}$$

In (4.3) we have

$$\begin{aligned} q_*=\int _0^{a_m} b(a) {{\bar{I}}}_*(a)\,\textrm{d}a=\kappa _1\left( 1-{\textsf{r}}_0 \right) \end{aligned}$$

so that

$$\begin{aligned} A_1^*=\Delta _N-\kappa _1{\textsf{r}}_0 \end{aligned}$$

and

$$\begin{aligned} P_*I={\textsf{r}}_0 \kappa _2 \int _0^{a_m}b(a)I(a)\,\textrm{d}a. \end{aligned}$$

In the following we still assume $p>(2\vee n)$.

#### Proposition 6.2

Assume  (2.4) and (2.5). For $1<{\textsf{R}}_0 <3$, the endemic steady state $({{\bar{S}}}_*,{{\bar{I}}}_*)$ to (1.1) is linearly stable in $L_p(\Omega )\times L_1(J,L_p(\Omega ))$.

#### Proof

Let $\lambda$ be a spectral point of the linearization, so that, according to Remark 4.5, there is a nontrivial $(S,I)\in W_{p,N}^{2}(\Omega )\times C(J,L_p(\Omega ))$ with

$$\begin{aligned} \left( \begin{matrix} A_1^* &{} -P_* \\ 0&{} -\partial _a+A(a) \end{matrix}\right) \left( \begin{matrix} S \\ I \end{matrix}\right) =\lambda \left( \begin{matrix} S \\ I \end{matrix}\right) \,,\qquad I(0)=P_*I +q_*S\,. \end{aligned}$$

That is,

6.11a $$\begin{aligned} -\Delta _NS&=-(\lambda +\kappa _1{\textsf{r}}_0 ) S-{\textsf{r}}_0 \kappa _2\int _0^{a_m}b(a)I(a)\,\textrm{d}a\,, \end{aligned}$$

6.11b $$\begin{aligned} \partial _aI(a)&=\big (-\lambda +A(a)\big )I(a) \,,\qquad a\in J\,, \end{aligned}$$

6.11c $$\begin{aligned} I(0)&={\textsf{r}}_0 \kappa _2\int _0^{a_m}b(a)I(a)\,\textrm{d}a+\kappa _1\left( 1-{\textsf{r}}_0 \right) S\,. \end{aligned}$$

From (6.11b) we get

$$\begin{aligned} I(a)=U_A^\lambda (a,0)I(0),\quad a\in J, \end{aligned}$$

and plugged into (6.11c) this yields

6.12 $$\begin{aligned} I(0)={{\bar{S}}}_*Q^\lambda I(0) +\kappa _1\left( 1-{\textsf{r}}_0 \right) S \end{aligned}$$

with

6.13 $$\begin{aligned} {{\bar{S}}}_*Q^\lambda ={\textsf{r}}_0 \kappa _2\int _0^{a_m} b(a) U_A^\lambda (a,0)\,\textrm{d}a\in \mathcal {L}\big (L_p(\Omega ),W_{p,N}^{1}(\Omega )\big )\,. \end{aligned}$$

In order to verify that $\textrm{Re}\,\lambda <0$, we assume for contradiction that $\textrm{Re}\,\lambda \ge 0$. Then $\lambda +\kappa _1{\textsf{r}}_0 -\Delta _N$ is invertible and we infer from (6.11a) and (6.12) that

6.14 $$\begin{aligned} (1-{{\bar{S}}}_*Q^\lambda ) I(0) =-\kappa _1\left( 1-{\textsf{r}}_0 \right) \big (\lambda +\kappa _1{\textsf{r}}_0 -\Delta _N\big )^{-1}S_*Q^\lambda I(0)\,. \end{aligned}$$

Recall that the eigenfunctions $(\phi _j)_{j\in \mathbb {N}}$ of the Neumann-Laplacian, corresponding to the eigenvalues counted according to multiplicity, build an orthonormal basis in $W_{2,N}^1(\Omega )$. Then $-\Delta _N\phi _j=\mu _j \phi$ entails $e^{t\Delta _N}\phi _j=e^{-t\mu _j}\phi _j$ for $t\ge 0$, and the operator ${{\bar{S}}}_*Q^\lambda$ leaves the eigenfunctions invariant. More precisely, from (6.1) we deduce

$$\begin{aligned}{} & {} {{\bar{S}}}_*Q^\lambda \phi _j =\mathcal {R}_{\lambda ,\mu _j}\phi _j, \nonumber \\ {}{} & {} \mathcal {R}_{\lambda ,\mu _j}:={\textsf{r}}_0 \kappa _2\int _0^{a_m} b(a)\,\Pi (a)\, e^{-\lambda a}\,\exp \left( -\mu _j\int _0^ad(\sigma )\,\textrm{d}\sigma \right) \,\textrm{d}a, \end{aligned}$$

for every $j\in \mathbb {N}$. Note that $I(0)\in W_{p,N}^1(\Omega )\hookrightarrow W_{2,N}^1(\Omega )$ is nonzero as otherwise also $S=0$. Hence, writing $I(0)=\sum _j \xi _j\phi _j$, we derive from the identity (6.14) that

$$\begin{aligned} \left( 1-\mathcal {R}_{\lambda ,\mu _j}\right) \xi _j=-\frac{\kappa _1\left( 1-{\textsf{r}}_0 \right) }{\lambda +\kappa _1{\textsf{r}}_0 +\mu _j} \mathcal {R}_{\lambda ,\mu _j}\xi _j,\quad j\in \mathbb {N}. \end{aligned}$$

Taking any $j\in \mathbb {N}$ with $\xi _j\not =0$, the previous identity leads to the characteristic equation

6.15 $$\begin{aligned} \frac{1}{\mathcal {R}_{\lambda ,\mu _j}}= 1-\frac{1-{\textsf{r}}_0 }{\frac{\lambda +\mu _j}{\kappa _1}+{\textsf{r}}_0 }\,. \end{aligned}$$

Owing to $\mu _j\ge 0$ we have

6.16 $$\begin{aligned} \vert \mathcal {R}_{\lambda ,\mu _j}\vert \le \mathcal {R}_{\textrm{Re}\,\lambda ,0}\le \mathcal {R}_{0,0}={\textsf{r}}_0 {\textsf{R}}_0 =1\,,\qquad \textrm{Re}\,\lambda \ge 0\,. \end{aligned}$$

Clearly, (6.15) has no real solution $\lambda \ge 0$ since in this case

$$\begin{aligned} 0<\mathcal {R}_{\lambda ,\mu _j}\le 1,\qquad \frac{1-{\textsf{r}}_0 }{\frac{\lambda +\mu _j}{\kappa _1}+{\textsf{r}}_0 }>0. \end{aligned}$$

For an arbitrary $\lambda \in \mathbb {C}$ with $\textrm{Re}\,\lambda \ge 0$ we write

$$\begin{aligned} \zeta :=\frac{\lambda +\mu _j}{\kappa _1}=\alpha +i\beta ,\qquad \alpha \ge 0,\quad \beta \in \mathbb {R}, \end{aligned}$$

and obtain from (6.15) and (6.16) the contradiction

$$\begin{aligned} 1&\le \frac{1}{\vert \mathcal {R}_{\lambda ,\mu _j}\vert ^2}= \left| 1-\frac{1-{\textsf{r}}_0 }{\zeta +{\textsf{r}}_0 }\right| ^2 =\frac{\vert \zeta +2{\textsf{r}}_0 -1\vert ^2}{\vert \zeta +{\textsf{r}}_0 \vert ^2}=1+\frac{({\textsf{r}}_0 -1)(2\alpha +3{\textsf{r}}_0 -1)}{(\alpha +{\textsf{r}}_0 )^2+\beta ^2}<1 \end{aligned}$$

since ${\textsf{r}}_0 -1<0$ and $2\alpha +3{\textsf{r}}_0 -1\ge 3{\textsf{r}}_0 -1>0$ by our assumption that $1/3<{\textsf{r}}_0 <1$. Therefore, we conclude that indeed $\textrm{Re}\,\lambda <0$. Consequently, the endemic steady state $({{\bar{S}}}_*,{{\bar{I}}}_*)$ is linearly stable when $1<{\textsf{R}}_0 <3$. $\square$

Clearly, one expects $({{\bar{S}}}_*,{{\bar{I}}}_*)$ to be linearly stable for all ${\textsf{R}}_0 >1$.

## Appendix

### Regularity of *I*

We provide the missing step from the proof of Proposition 3.5.

#### Lemma 7.1

For $\varepsilon >0$ small and some $n/2p<2\theta <2-n/p$, let

$$\begin{aligned}{} & {} S_\varepsilon \in C\big ([0, T_m-\varepsilon ),W_{p,\mathcal {B}}^{2}(\Omega )\big ),\quad I_\varepsilon \in C\big ([0, T_m-\varepsilon ),L_1(J,W_{p,\mathcal {B}}^{2\theta }(\Omega ))\big ),\\{} & {} \qquad I_{0,\varepsilon }\in L_1\big (J,W_{p,\mathcal {B}}^{2\theta }(\Omega )\big ) \end{aligned}$$

be as in the proof of Proposition 3.5. Then

$$\begin{aligned} I_\varepsilon \in C\big ((0,T_m-\varepsilon ),L_1(J,W_{p,\mathcal {B}}^{2}(\Omega ))\big ),\qquad I\in C\big ((0,T_m),L_1(J,W_{p,\mathcal {B}}^{2}(\Omega ))\big ). \end{aligned}$$

#### Proof

We proceed analogously to the proof of Proposition 3.2. According to Lemma 3.1 there is $\alpha >0$ such that

$$\begin{aligned} B[S_\varepsilon ,I_\varepsilon ] \in C\big ([0, T_m-\varepsilon ),W_{p,\mathcal {B}}^{2\alpha }(\Omega )\big ) \end{aligned}$$

and we obtain from (3.5), for $0<t_2\le t_1\le T<T_m-\varepsilon$,

$$\begin{aligned}&||I_\varepsilon (t_1,\cdot )-I_\varepsilon (t_2,\cdot )\Vert _{L_1(J,W_{p,\mathcal {B}}^{2}(\Omega ))}\\&\le \int _0^{t_2}\Vert U_A(a,0)\Vert _{\mathcal {L}(W_{p,\mathcal {B}}^{2\alpha }(\Omega ),W_{p,\mathcal {B}}^{2}(\Omega ))}\, \Vert B[S_\varepsilon ,I_\varepsilon ](t_1-a) -B[S_\varepsilon ,I_\varepsilon ](t_2-a)\Vert _{W_{p,\mathcal {B}}^{2\alpha }(\Omega )}\,\textrm{d}a\\&\quad +\int _{t_2}^{t_1}\Vert U_A(a,0)\Vert _{\mathcal {L}(W_{p,\mathcal {B}}^{2\alpha }(\Omega ),W_{p,\mathcal {B}}^{2}(\Omega ))}\, \Vert B[S_\varepsilon ,I_\varepsilon ](t_1-a)\Vert _{W_{p,\mathcal {B}}^{2\alpha }(\Omega )}\,\textrm{d}a\\&\quad + \int _{t_2}^{t_1}\Vert U_A(a,a-t_2)\Vert _{\mathcal {L}(W_{p,\mathcal {B}}^{2\theta }(\Omega ),W_{p,\mathcal {B}}^{2}(\Omega ))}\, \Vert I_{0,\varepsilon }(a-t_2)\Vert _{W_{p,\mathcal {B}}^{2\theta }(\Omega )}\,\textrm{d}a\\&\quad + \int _{t_1}^{a_m}\big \Vert \big (U_A(a,a-t_1)-U_A(a,a-t_2)\big ) I_{0,\varepsilon }(a-t_1) \Vert _{W_{p,\mathcal {B}}^{2}(\Omega )}\,\textrm{d}a\\&\quad + \int _{t_1}^{a_m} \Vert U_A(a,a-t_2)\Vert _{\mathcal {L}(W_{p,\mathcal {B}}^{2\theta }(\Omega ),W_{p,\mathcal {B}}^{2}(\Omega ))}\, \Vert I_{0,\varepsilon }(a-t_1) -I_{0,\varepsilon }(a-t_2)\Vert _{W_{p,\mathcal {B}}^{2\theta }(\Omega )}\,\textrm{d}a\\&\le Me^\varpi \int _0^{t_2} a^{\alpha -1}\,\Vert B[S_\varepsilon ,I_\varepsilon ](t_1-a)-B[S_\varepsilon ,I_\varepsilon ](t_2-a) \Vert _{W_{p,\mathcal {B}}^{2\alpha }(\Omega )}\,\textrm{d}a\\&\quad +c(R) \int _{t_2}^{t_1}a^{\alpha -1}\,\textrm{d}a + t_2^{\theta -1}\int _{t_2}^{t_1} \Vert I_{0,\varepsilon }(a-t_2)\Vert _{W_{p,\mathcal {B}}^{2\theta }(\Omega )}\,\textrm{d}a\\&\quad + \int _{t_1}^{a_m}\big \Vert \big (U_A(a,a-t_1)-U_A(a,a-t_2)\big ) I_{0,\varepsilon }(a-t_1) \Vert _{W_{p,\mathcal {B}}^{2}(\Omega )}\,\textrm{d}a\\&\quad + t_2^{\theta -1} \int _{t_1}^{a_m} \Vert I_{0,\varepsilon }(a-t_1)-I_{0,\varepsilon }(a-t_2)\Vert _{W_{p,\mathcal {B}}^{2\theta }(\Omega )}\,\textrm{d}a\,. \end{aligned}$$

Now, as $\vert t_1-t_2\vert \rightarrow 0$, the first integral on the right-hand side goes to zero since the function $B[S_\varepsilon ,I_\varepsilon ]\in C([0,T],W_{p,\mathcal {B}}^{2\alpha }(\Omega ))$ is uniformly continuous while the second and the third integral vanish since $a\mapsto a^{\alpha -1}$ respectively $I_{0,\varepsilon }$ are integrable. To see that the fourth integral vanishes in the limit one may use the strong continuity (Amann 1995, Equation II. (2.1.2)) of the evolution operator $U_A$ in $\mathcal {L}\big (W_{p,\mathcal {B}}^{2\theta }(\Omega ),W_{p,\mathcal {B}}^{2}(\Omega )\big )$ and Lebesgue’s theorem. For the last integral one may use the strong continuity of the translations on $L_1\big (J,W_{p,\mathcal {B}}^{2\theta }(\Omega )\big )$. Consequently, $I_\varepsilon \in C\big ((0,T_m-\varepsilon ),L_1(J,W_{p,\mathcal {B}}^{2}(\Omega ))\big )$. Letting $\varepsilon \rightarrow 0$ yields $I\in C\big ((0,T_m),L_1(J,W_{p,\mathcal {B}}^{2}(\Omega ))\big )$. $\square$

### Proof of the $L_1$-inequality (3.16)

We derive inequality (3.16). To this end, note from Gauss’ theorem that

7.1 $$\begin{aligned} \int _\Omega \Delta u\,\textrm{d}x=\int _{\partial \Omega } \partial _\nu u\,\textrm{d}\sigma \le 0,\qquad u\in W_{p,\mathcal {B}}^2(\Omega ),\quad u\ge 0, \end{aligned}$$

since $\partial _\nu u=0$ on $\partial \Omega$ if $\delta =1$ and $\partial _\nu u\le 0$ on $\partial \Omega$ if $\delta =0$. Now, for $a\in J$ fixed set

$$\begin{aligned} w(t):=U_A(a+t,a)I_0(a),\quad t\in [0,a_m-a]. \end{aligned}$$

Integrating then

$$\begin{aligned} \frac{\textrm{d}}{\textrm{d}t} w(t)=A(a+t)w(t),\quad t\in (0,a_m-a], \end{aligned}$$

with *A* given in (2.1), we get from (7.1)

$$\begin{aligned} \int _\Omega w(t,x)\,\textrm{d}x\le \int _\Omega w(0,x)\,\textrm{d}x-\int _0^t\int _\Omega \big (m(a+\tau ,x)+r(a+\tau ,x)\big )\, w(\tau ,x)\,\textrm{d}x\,\textrm{d}\tau \,, \end{aligned}$$

and therefore

$$\begin{aligned}&\int _\Omega U_A(a+t,a)I_0(a)\,\textrm{d}x \nonumber \\&\le \int _\Omega I_0(a)\,\textrm{d}x-\int _0^t\int _\Omega \big (m(a+\tau )+r(a+\tau )\big )\, U_A(a+\tau ,a)I_0(a)\,\textrm{d}x\,\textrm{d}\tau \,. \end{aligned}$$

Similarly, one derives for $t>a$ that

$$\begin{aligned} \int _\Omega U_A(&t-a,0)I(a,0)\,\textrm{d}x\\&\le \int _\Omega I(a,0)\,\textrm{d}x-\int _a^t\int _\Omega \big (m(\tau -a)+r(\tau -a)\big )\, U_A(\tau -a,0)I(a,0)\,\textrm{d}x\,\textrm{d}\tau \,. \end{aligned}$$

We then recall (3.14) and use the previous two identities to obtain

$$\begin{aligned} \int _0^{a_m}\int _\Omega I(t,a)\,\textrm{d}x\,\textrm{d}a&= \int _0^t\int _\Omega U_A(t-a,0) I(a,0)\,\textrm{d}x\textrm{d}a+\int _0^{a_m-t}\int _\Omega U_A(a+t,a)I_0(a)\,\textrm{d}x\,\textrm{d}a\\&\le \int _0^t\int _\Omega I(a,0)\,\textrm{d}x\,\textrm{d}a +\int _0^{a_m-t}\int _\Omega I_0(a)\,\textrm{d}x\,\textrm{d}a\\&\quad - \int _0^t\int _a^t\int _\Omega \big (m(\tau -a)+r(\tau -a)\big )\, U_A(\tau -a,0)I(a,0)\,\textrm{d}x\,\textrm{d}\tau \,\textrm{d}a\\&\quad -\int _0^{a_m-t}\int _0^t\int _\Omega \big (m(a+\tau )+r(a+\tau )\big )\, U_A(a+\tau ,a)I_0(a)\,\textrm{d}x\,\textrm{d}\tau \,\textrm{d}a\\&=\int _0^t\int _\Omega I(a,0)\,\textrm{d}x\,\textrm{d}a +\int _0^{a_m}\int _\Omega I_0(a)\,\textrm{d}x\,\textrm{d}a\\&\quad - \int _0^t\int _0^\tau \int _\Omega \big (m(a)+r(a)\big )\, U_A(a,0)I(\tau -a,0)\,\textrm{d}x\,\textrm{d}a\,\textrm{d}\tau \\&\quad -\int _0^t\int _\tau ^{a_m}\int _\Omega \big (m(a)+r(a)\big )\, U_A(a,a-\tau )I_0(a-\tau )\,\textrm{d}x\,\textrm{d}a\,\textrm{d}\tau \\&\quad - \int _{a_m-t}^{a_m}\int _\Omega I_0(a)\,\textrm{d}x\,\textrm{d}a\\&\quad + \int _0^t\int _{a_m-t+\tau }^{a_m}\int _\Omega \big (m(a)+r(a)\big )\, U_A(a,a-\tau )I_0(a-\tau )\,\textrm{d}x\,\textrm{d}a\,\textrm{d}\tau \,. \end{aligned}$$

Using (3.14) again we may rewrite the previous inequality as

7.2 $$\begin{aligned} \begin{aligned} \int _0^{a_m}\int _\Omega I(t,a)\,\textrm{d}x\,\textrm{d}a&\le \int _0^{a_m}\int _\Omega I_0(a)\,\textrm{d}x\,\textrm{d}a +\int _0^t\int _\Omega I(\tau ,0)\,\textrm{d}x\,\textrm{d}\tau \\&\quad - \int _0^t\int _0^{a_m}\int _\Omega \big (m(a)+r(a)\big )\,I(\tau ,a)\,\textrm{d}x\,\textrm{d}a\,\textrm{d}\tau \\&\quad - \int _{a_m-t}^{a_m}\int _\Omega I_0(a)\,\textrm{d}x\,\textrm{d}a\\&\quad + \int _0^t\int _{a_m-t+\tau }^{a_m}\int _\Omega \big (m(a)+r(a)\big )\, U_A(a,a-\tau )I_0(a-\tau )\,\textrm{d}x\,\textrm{d}a\,\textrm{d}\tau . \end{aligned} \end{aligned}$$

Integrating (1.1c) and using (7.1) yields

7.3 $$\begin{aligned} \int _\Omega S(t)\,\textrm{d}x\le & {} \int _\Omega S_0\,\textrm{d}x+\int _0^{a_m}\int _\Omega I_0(a)\,\textrm{d}x\,\textrm{d}a+\int _0^t\int _\Omega \kappa _1\left( 1-\frac{S(\tau )}{\kappa _2}\right) S(\tau )\,\textrm{d}x\,\textrm{d}\tau \nonumber \\{} & {} -\int _0^t\int _\Omega I(\tau ,0)\,\textrm{d}x\,\textrm{d}\tau +\int _0^t\int _\Omega \int _0^{a_m} r(a) I(\tau ,a)\,\textrm{d}a\,\textrm{d}x\,\textrm{d}\tau . \end{aligned}$$

Adding (7.2) and (7.3) yields (3.16).

#### Remark 7.2

When considering Neumann boundary conditions ($\delta =1$), then (7.2) and (7.3) are equalities and thus also (3.16).

## Footnotes

1 Here and in the following, if $t>a_m$, then integrals $\int _0^t\textrm{d}a$ equal $\int _0^{a_m}\textrm{d}a$ and integrals $\int _0^{a_m-t}\textrm{d}a$ vanish.

2 We recall again that for $t>a_m$, integral $\int _0^t\textrm{d}a$ equal $\int _0^{a_m}\textrm{d}a$ and integrals $\int _0^{a_m-t}\textrm{d}a$ vanish.
